# Enteroendocrine cell differentiation: Implications for human disease

**DOI:** 10.1016/j.mce.2025.112607

**Published:** 2025-06-26

**Authors:** Elisa Saint-Denis, Bianca Frintu, Madelyn Goldsmith, Guilherme P. Ramos, Daniel Zeve

**Affiliations:** a Division of Endocrinology, Boston Children’s Hospital, Boston, MA, 02115, USA; b Division of Gastroenterology, Department of Internal Medicine, Mayo Clinic, Phoenix, AZ, 85054, USA; c Department of Pediatrics, Harvard Medical School, Boston, MA, 02115, USA

## Abstract

Enteroendocrine cells are the most abundant hormone producing cells in humans. Though they make up less than 1 % of the gastrointestinal epithelium, these cells have a large physiological impact through the secretion of hormones that act both locally and systemically to regulate intestinal function and whole-body metabolism, among other functions. The differentiation of enteroendocrine cells from intestinal stem cells is complex, involving not only lineage, but hormonal specification. This review highlights the specific signaling pathways and transcription factors that regulate enteroendocrine cell differentiation and hormone production, integrating newer findings into our growing understanding of this process. Further, it also describes how enteroendocrine cells and their differentiation are involved and altered in human health and disease: specifically aging, inflammatory bowel disease, obesity, and diabetes mellitus. Finally, we focus on how enteroendocrine cells can be targeted to produce insulin, a growing field with significant implications. Understanding what drives enteroendocrine differentiation, both molecularly and physiologically, will provide important insights into how these cells can serve as future therapeutic targets.

## 1. Introduction

We are currently in the midst of a medical revolution targeting obesity and type 2 diabetes mellitus (T2DM). At the center of this is glucagon-like peptide 1 (GLP1) and glucose-dependent insulinotropic peptide (GIP) receptor agonists, such as the GLP1 receptor agonists semaglutide and dulaglutide, and the GIP/GLP1 receptor agonist tirzepatide, which have changed the way physicians treat these diseases and their negative sequelae. The popularity of these medications has put a spotlight on enteroendocrine (EE) cells, the hormone-producing cells of the gastrointestinal (GI) tract that produce much more than GIP and GLP1.

EE cells comprise approximately 1 % of the GI epithelium, making them one of the rarest cell populations within the intestine (Nwako and McCauley, 2024; Sanchez et al., 2022). However, due to the extensive size of the gut, their presence makes the GI tract the largest endocrine organ in the body. To elaborate on this, the pancreas is the second largest endocrine organ with 1.4×10^10^ endocrine cells. (Da Silva Xavier, 2018; Pisania et al., 2010). In comparison, the adult human intestine has roughly 1.6×10^12^ epithelial cells. (Gespach, 2012; Helander and Fandriks, 2014). As EE cells make up for 1 % of the intestinal epithelium, this would mean that the GI tract has 1.6×10^10^ EE cells, two billion more endocrine cells than the pancreas.

Throughout the GI tract, EE cells produce at least 25 different hormones that function to regulate local GI function, whole body metabolism, and multiple other aspects of human health (Gribble and Reimann, 2019). In the setting of rapid cell turnover, EE cell numbers and hormone production are maintained through intestinal stem cell (ISC) differentiation. Through a complex integration of signaling pathways and transcription factors, ISCs differentiate into multiple EE cell types. As our understanding of the requirements for EE cell differentiation expands, so does our understanding of how EE cell function is implicated in the pathogenesis of multiple disease states.

This review integrates studies conducted in a wide range of models and systems, with key differences between models occasionally arising. These differences may be secondary to the unique biological contexts of each model that may be difficult to replicate in tissue culture. Throughout, we aim to highlight where these model-specific differences are relevant to provide a comprehensive overview of specific pathways and factors involved in mammalian EE cell differentiation and hormone production, as well as how disruption of EE cell differentiation can impact human health.

## 2. Enteroendocrine cell types

**Fig. 1.**
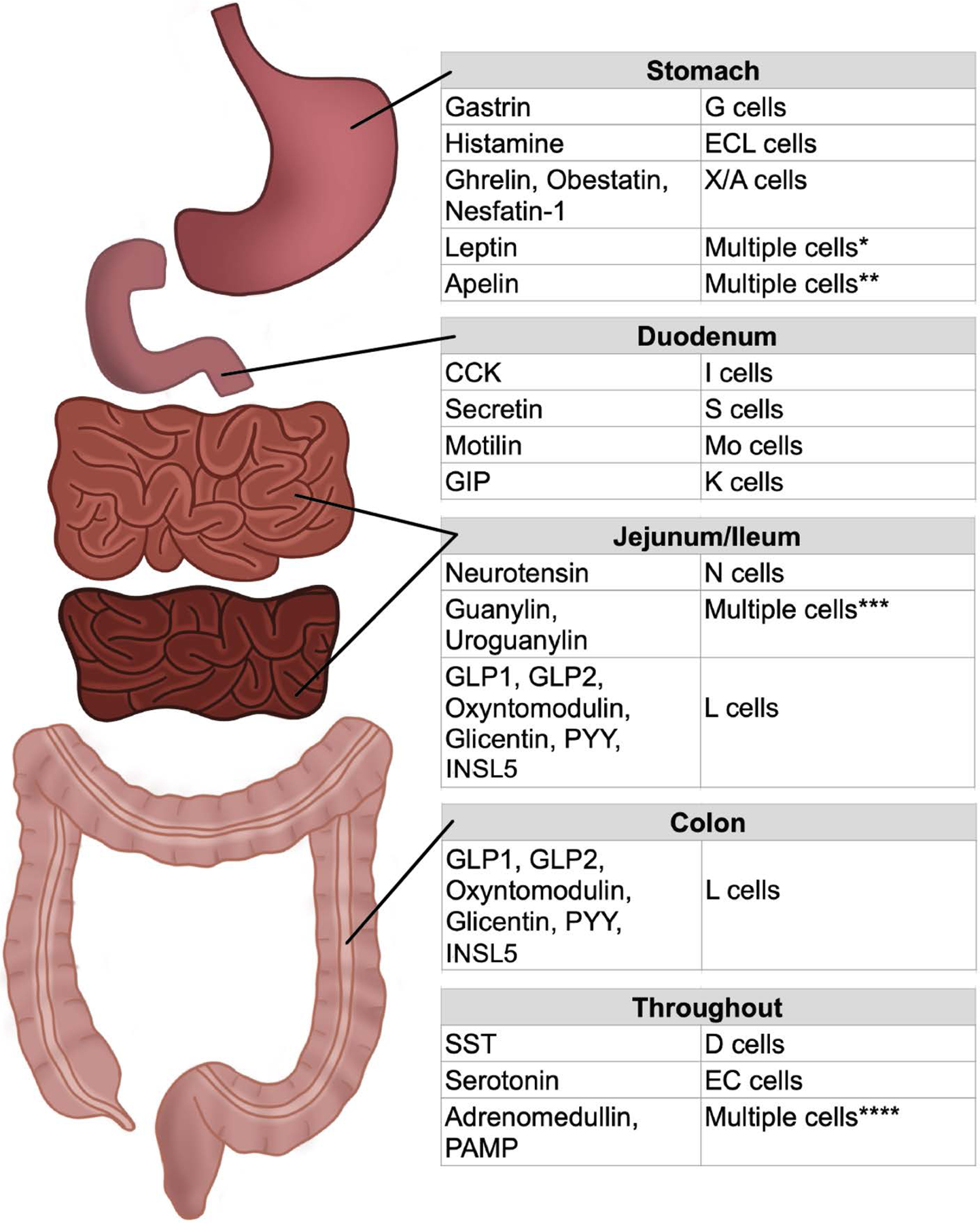
Enteroendocrine Cells and the GI Tract Enteroendocrine (EE) cell types, the hormones they produce, and their location in the GI tract. *Leptin is produced by chief cells and endocrine P cells; **Apelin is produced by parietal cells, chief cells, mucous neck cells, and multiple EE cell types; ***Guanylin/uroguanylin are produced by enterochromaffin (EC) cells, enterocytes, goblet cells, and Paneth cells; ****Adrenomedullin and PAMP are produced by multiple EE cell types and chief cells.

EE cells represent a very diverse cell type, with at least 25 different intestinal hormones, each being produced by a specific mature cell in response to stimuli. The EE cell lineage is complex, as these cells, and the hormones they produce, are also not homogeneously distributed throughout the GI tract. In one human study, it was noted that even though the gene expression of chromogranin A (CHGA), a marker for EE cells, was expressed at similar levels throughout the entire intestinal tract (the stomach was not evaluated), CHGA protein was highest in the duodenum and rectum (Jorsal et al., 2018), suggesting those sites have a higher concentration of EE cells than other parts of the GI tract. Further, while it is well known that certain hormones are predominantly made in specific areas of the intestine (proximal vs distal small intestine vs. large intestine), select hormones are more broadly produced in multiple areas of the gut (Atanga et al., 2023; Gunawardene et al., 2011). For example, among the more ubiquitously expressed hormones, somatostatin (SST, produced by D cells) and serotonin (5-HT, produced by enterochromaffin (EC) cells), are expressed throughout the GI tract and function to regulate GI motility and maintain metabolic homeostasis (Banskota et al., 2019; Mawe and Hoffman, 2013; Shamsi et al., 2021; Tulassay, 1998). Two additional hormones, adrenomedullin and proadrenomedullin N-terminal 20 peptide (PAMP), are expressed by EE cells and non-EE cells throughout the GI tract, and regulate gastric function, intestinal contractility, nutrient absorption, and insulin secretion (Martinez-Herrero and Martinez, 2022). EE cell types, the main hormones they produce, and their most predominant location in the GI tract are summarized in Fig. 1. Notably, EE cell nomenclature has been the subject of debate over the past decade, and there has been calls for updating the classification system based on main hormone produced and location, among other factors (Fothergill and Furness, 2018; Helander and Fandriks, 2012). For the purposes of this review, we will be using the current EE cell naming scheme, focusing on the main hormone produced by each cell type.

Other hormones are more specific to the stomach, including gastrin (produced by G cells) and histamine (produced by enterochromaffin-like (ECL) cells), both of which function in digestion (Atanga et al., 2023). There are multiple appetite-regulating hormones that are produced by X/A cells, including: 1) acylated ghrelin, which serves as an orexigenic signal; 2) unacylated ghrelin, which acts in opposition to acylated ghrelin; 3) obestatin, which, similar to unacylated ghrelin, acts as an anorexigenic signaling peptide and is produced from the proghrelin peptide, and 4) nesfatin-1, which also functions as an anorexigenic hormone (Akalu et al., 2020; Schalla and Stengel, 2018; Stengel and Tache, 2012; Villarreal et al., 2022). The stomach also produces leptin from primarily chief cells, but also endocrine P cells, and acts to regulate intestinal function and satiety (Cammisotto and Bendayan, 2012; Guilmeau et al., 2004). Apelin is also expressed predominantly in the stomach and is associated with both exocrine and EE cells. It plays a role regulating multiple GI functions, including gastric acid secretion, appetite, and secretion of the EE cell hormone cholecystokinin (CCK) (Huang et al., 2019).

CCK is produced by I cells of primarily the duodenum and jejunum and functions to induce bile acid secretion, pancreatic enzyme release, insulin secretion, and appetite suppression (Rehfeld, 2017). Secretin (SCT) is produced from primarily duodenal S cells, where SCT protein expression is highest, but is found at lower levels throughout the rest of the small intestine (Gilliam-Vigh et al., 2023). It functions to regulate intestinal pH, as well as other gastric functions (Laurila et al., 2021). The human specific EE hormone motilin (MLN) is produced by Mo cells of the duodenum and jejunum and regulates multiple GI processes, including motility, as well as possibly regulating glucose homeostasis and hunger (Deloose et al., 2019). GIP is produced by K cells of the small intestine, which are predominantly found in the duodenum. GIP is one of the incretin hormones that play critical roles in insulin production/secretion and appetite suppression after oral nutrient intake. Along with its incretin function, GIP is also involved in reducing gastric acid secretion (Guccio et al., 2022; Seino et al., 2010). Neurotensin, secreted by N cells primarily in the jejunum and ileum, is largely induced secondary to fat ingestion and regulates GI function and nutrient homeostasis (Kalafatakis and Triantafyllou, 2011). Two additional hormones, guanylin (GN, primarily produced in the large intestine) and uroguanylin (UGN, primarily expressed in the small intestine), function to regulate intestinal fluid homeostasis and satiety (UGN specific). Multiple intestinal cells produce GN and UGN; some studies show they are produced by EC cells while others show that non-EE intestinal cells make them, and that some of these differences may be species specific (Cetin et al., 1994; Dye et al., 2019; Folgueira et al., 2018; Ikpa et al., 2016; Sindic, 2013).

One additional EE cell is the L cell, which is found in both the small and large intestines (predominantly in the ileum and rectum) and produces up to seven different hormones. Through cleavage of proglucagon, L cells can produce GLP1 (the other incretin hormone), GLP2, oxyntomodulin, glicentin, and glicentin-related pancreatic peptide (GRPP). Along with these hormones, L cells also produce peptide YY (PYY) and insulin-like peptide 5 (INSL5). Aside from regulating GI function, GLP1, oxyntomodulin, glicentin, and PYY also decrease appetite and regulate insulin secretion; GLP2 does not alter appetite or insulin but regulates intestinal barrier function and blood flow while the physiological function of intestinal GRPP is not known (Kuhre et al., 2021; Lafferty et al., 2021; Spreckley and Murphy, 2015). The role of INSL5 is also unclear, but it possibly plays a role in colon function and appetite (Diwakarla et al., 2020; Lewis et al., 2020; Zaykov et al., 2019).

This diversity of EE cells suggests that each mature cell only produces a specific hormone or set of hormones. Data over the last 15 years, however, from both mice and humans, suggest that mature EE cells are more permissive in the hormones they produce. In studies using the mouse GI tract, GIP-positive K cells also expressed mRNA transcripts for *Cck, Gcg*, *Pyy*, *Sct*, and *Sst*, while small intestinal glucagon (GCG)-positive L cells also expressed mRNA transcripts for *Cck*, *Gip*, *Nts*, *Pyy*, and *Sct*. Flow cytometric analysis supported these findings, with 15 % of small intestinal L cells also producing GIP and almost all small intestinal L cells producing CCK. In a specific subset of K cells, co-expression with CCK was common while a minority of K cells co-expressed GLP1 and SST (Habib et al., 2012). Additional murine studies showed that in the stomach, 5-HT was produced in the histamine-producing ECL cells (Koo et al., 2021). In fact, many other murine studies have shown co-expression of 5-HT with other hormones, including CCK, SCT, GHRL, GLP1, as well as combinations of these hormones with and without 5-HT (Diwakarla et al., 2017; Fothergill et al., 2017). One study even identified a small intestinal EE cell that expressed CCK, GIP, GLP1, NTS, PYY and SCT (Egerod et al., 2012). Further, analysis of the human Gut Cell Atlas showed that *SCT* was expressed in every EE cell subtype at similar levels, suggesting that S cells, which predominantly produce SCT, may not be a distinct entity (Hysenaj et al., 2024), although it is possible that one specific cell produces and secretes SCT while all others merely express the gene. Notably, these findings are distinct from a previous study that shows decreasing *SCT* expression along the small intestine, with no expression in the large intestine (Gilliam-Vigh et al., 2023). Additionally, examining EE cells within human jejunum showed multiple varieties of EE hormone co-expression, including GIP/CCK/proglucagon peptides, GHRL/MLN, and 5-HT with GIP, GHRL, PYY, or SCT (Fazio Coles et al., 2020). These data show that EE hormone production is not as simple as “one cell, one hormone; ” however, the physiological relevance of these hormone combinations is not known. Below, we will continue to use the lettered nomenclature to describe the predominant hormone produced from specific EE cells.

## 3. Transcription factors regulating enteroendocrine differentiation

**Fig. 2.**
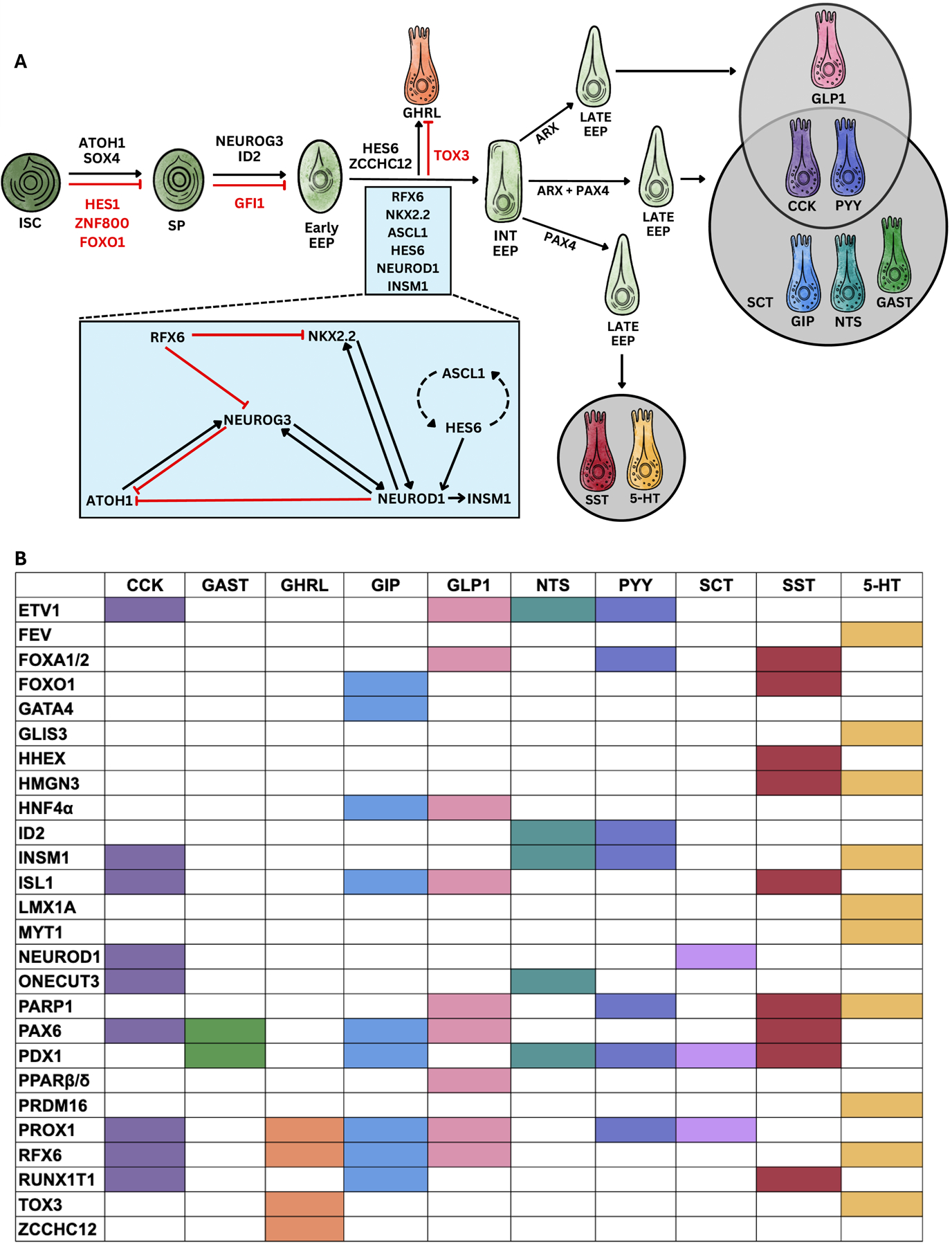
Transcription Factors Regulating Enteroendocrine Differentiation (a) Transcription factors regulating enteroendocrine differentiation, highlighting factors important for the transition from intestinal stem cell (ISC) to secretory progenitor (SP) and then from SP to early, intermediate and late enteroendocrine progenitors (EEPs). Transcription factors in red inhibit differentiation and transcription factors in black promote it. Differential expression of ARX and PAX4 specifies three late EEP types. The insert highlights the complicated transcription factor interactions present during differentiation from the SP to early EEP based on previous studies. This figure compiles data from various murine and human models at various stages across the lifespan. (b) Table of transcription factors involved in specific hormone production in late stage enteroendocrine differentiation. Shading indicates that the specific transcription factor functions to regulate the expression of a particular hormone.

While there are multiple exogenous factors that regulate hormone expression and function, including GI location, nutrient availability, and microbiome profile, here we will focus on the transcriptional regulators that drive EE cell lineage differentiation and hormone production in GI tissues. All mature intestinal epithelial cells are derived from multi-potent LGR5-positive ISCs (reviewed in Barker et al., 2007; Beumer and Clevers, 2021; Kolev and Kaestner, 2023). ISCs then differentiate into two different lineages, the absorptive lineage (enterocytes), which function to absorb nutrients, and the secretory lineage, which includes goblet cells, Paneth cells, tuft cells, and EE cells (Beumer and Clevers, 2021; Kolev and Kaestner, 2023). The transcription factors involved in differentiation from ISCs to late EE progenitors, as well as the relevant transcription factor interactions, are summarized in Fig. 2A. Of note, conclusions drawn in this section and compiled in Fig. 2A are made from results found in murine and human *in vivo*, *ex vivo*, and *in vitro* studies. Importantly, some results vary between the models used, which may reflect the distinct biological context they represent-some regulatory mechanisms may require interactions between multiple tissues and organs that cannot easily be replicated in tissue culture. Where model-specific differences are relevant, they will be highlighted accordingly.

Initial specification of the secretory lineage involves the suppression of hairy and enhancer of split 1 (HES1) and the subsequent activation of atonal bHLH transcription factor (ATOH1); it has been shown in multiple mouse studies that *Atoh1* deletion induces loss of all secretory cells, including EE cells, without altering enterocyte differentiation (Shroyer et al., 2007; Yang et al., 2001). In *Hes1* deletion studies, secretory lineage cells are increased postnatally but normalize into adulthood; it was shown that this was secondary to functional compensation by HES3 and HES5 after birth, with a triple knockout of *Hes1*/*3*/*5* increasing the number of secretory cells through two months of age (Jensen et al., 2000; Noah and Shroyer, 2013; Ueo et al., 2012).

All secretory cell types are derived from ATOH1-expressing progenitor cells. Therefore, subsequent expression of other key transcription factors directs progenitors to specific cell types: neurogenin-3 (NEUROG3) directs progenitors towards EE differentiation while growth factor independent 1 (GFI1), SAM pointed domain containing ETS (SPDEF), and SRY-box containing gene 9 (SOX9) direct goblet and Paneth differentiation (Bastide et al., 2007; Beumer and Clevers, 2021; Bjerknes and Cheng, 2010; Jenny et al., 2002; Kolev and Kaestner, 2023; Lee et al., 2002; Lo et al., 2017; Mori-Akiyama et al., 2007; Shroyer et al., 2005). However, secretory progenitor lineage allocation may be more complicated than this, with reciprocal interactions between ATOH1 and its downstream targets, as well as dosage effects, playing a role in determining secretory progenitor fate. One study found that isolated *Atoh1*-positive cells had no enrichment in EE cell genes, and that *Atoh1* is expressed at much lower levels in EE cells than in goblet cells and Paneth cells, suggesting that *Atoh1* expression is suppressed as cells enter the EE cell lineage (Lo et al., 2017). This same study found that *Spdef* is both a target of ATOH1 and functions as a transcriptional co-regulator of ATOH1, amplifying ATOH1 dependent transcription. Along with this, it has been previously shown that GFI1 functions to suppress *Neurog3* expression (Bjerknes and Cheng, 2010), suggesting that both SPDEF and GFI1 work to maintain ATOH1 expression and to suppress NEUROG3 expression, preventing EE cell differentiation.

Interestingly, one murine study compared gene expression after deleting one or both *Neurog3* alleles compared to controls in the small intestine. *Atoh1* expression was significantly higher in *Neurog3* cells with homozygous deletion than in *Neurog3* cells with hemizygous deletion; further, no *Atoh1* expression was detected in control cells. However, hemizygous cells had sufficiently decreased *Neurog3* levels to significantly reduce expression of EE-specific transcription factors, leading to expression of both goblet and EE cell lineage markers, suggesting *Neurog3* expression alone is not sufficient to limit secretory progenitors to the EE cell lineage (Li et al., 2021). Therefore, EE cells require higher expression of NEUROG3 along with lower levels of ATOH1, which is likely accomplished via a negative feedback loop between ATOH1 and NEUROG3. Other factors may also help increase expression of NEUROG3 in secretory progenitor cells. In the pancreas, several transcription factors, some that operate downstream in EE cell specification, have been implicated to amplify *Neurog3* expression, including NK2 Homeobox 2 (NKX2.2), Forkhead box protein A2 (FOXA2), and Glis family zinc finger 3 (GLIS3) (Churchill et al., 2017; Ejarque et al., 2013; Kim et al., 2012; Yang et al., 2011). However, it appears these factors likely do not directly regulate *Neurog3* expression in the intestine. One study found that intestinal *Neurog3* expression was not altered in Nkx2.2 knockout mice, another found *Neurog3* expression also unchanged in Foxa1/2 knockout mice jejunum, and no studies link *Glis3* to *Neurog3* expression in mammalian intestine (Desai et al., 2008; Ye and Kaestner, 2009).

Additionally, although ATOH1 is typically considered the main driver of secretory differentiation, SRY-box transcription factor 4 (SOX4) has also been reported to regulate this transition in an ATOH1-independent manner (Gracz et al., 2018). In *Sox4* knockout mice, both tuft and EE cell lineage differentiation was decreased, including cells expressing GIP, CCK, SST, GCG, PYY, and GLP2. Interestingly, enterochromaffin (EC) cell differentiation was not altered by *Sox4* deletion. These findings were also noted in murine intestinal organoid studies, which showed that in the setting of *Atoh1* deletion, overexpression of *Sox4* was able to rescue tuft and EE cell lineage differentiation. However, counter to the *in vivo* data, mRNA levels of *Cck* and *Gcg* were not rescued with *Sox4* overexpression, suggesting that ATOH1 is required for specific hormones to be expressed (Gracz et al., 2018). Further, Zinc finger protein 800 (ZNF800) was recently identified through a CRISPR screen as a master negative regulator of the EE cell lineage. Deletion of ZNF800 in human small intestinal organoids led to a marked increase in EE cell number, with a comparable decrease in goblet and Paneth cells. Further, it was shown that ZNF800 decreased expression of *SOX4*, suggesting that it works at, or upstream of, the level of the secretory progenitor cell (Lin et al., 2023).

Although NEUROG3 is known as the master regulator necessary for all EE cell differentiation (Cortina et al., 2007; Jenny et al., 2002; Lee et al., 2002; Nwako and McCauley, 2024; Sanchez et al., 2022), it may require additional transcription factors to induce the EE cell lineage differentiation. In fact, lineage tracing experiments in mice found *Neurog3* expression in 13–14 % of all goblet and Paneth cells in the duodenum (Schonhoff et al., 2004), suggesting that additional factors are necessary to drive EE cell differentiation. This includes the transcription factor neuronal differentiation 1 (NEUROD1). In one study using a transgenic mouse model, over expression of *Neurod1* in the intestinal epithelium induced increased expression of *Neurog3* and decreased expression of *Atoh1* and the Paneth cell markers *Dpp4* and *Lyz*, suggesting that NEUROD1 has an early role in EE cell differentiation (Li et al., 2019a). However, another study found that *Neurod1* knockout mice resulted in loss of CCK- and SCT-positive cells, while 5-HT-, PYY-, NTS-, GIP-, and SST-positive cells were unaffected, suggesting that NEUROD1 acts downstream of NEUROG3 (Naya et al., 1997). Additional transcription factors critical to EE cell differentiation were identified when using NEUROD1 as a marker of mature EE cells. Using a 2D-model of human EE cell differentiation, a recent study showed that both achaete-scute family bHLH transcription factor 1(ASCL1) and Hes family BHLH transcription factor 6 (HES6) marked EE progenitor cells that were downstream of NEUROG3, and these regulate each other to specify the timing and activation of NEUROD1. Through deletion studies, it was shown that ASCL1 is critical for EC development, HES6 is important for X/A cell development, and NEUROD1 drives peptide-hormone production (Singh et al., 2024). Another study examined ASCL1 function in gastric epithelium and noted that *Ascl1* null mice had a loss of almost all EE cell markers except for those signifying ECL cells. Notably, *Ascl1* expression in adult mice from this study was found in the stomach but was absent in the remainder of the GI tract (Hibdon et al., 2023).

Many other transcription factors act downstream of NEUROG3. Some of these are required for all EE cells, likely playing a role early in EE cell differentiation, while others are likely required during the later stages of EE cell differentiation. One such early transcription factor is NKX2–2, which is expressed with NEUROG3 and regulates NEUROD1 expression, suggesting a function early in differentiation (Desai et al., 2008; Li et al., 2019a; Wang et al., 2009). In fact, mouse knockout models showed a reduction of most EE cell populations, except for X/A cells, while maintaining all other secretory lineages (Desai et al., 2008). Aristaless-related homeobox (ARX) and paired box 4 (PAX4) are another set of transcription factors that further subdivide the EE cell lineage. Knockout studies in secretory progenitor cells have revealed the specification of some EE cells rely on both *Arx* and *Pax4* expression, while others depend only on *Arx* or *Pax4*. *Arx*-deficient mice have a reduction or complete loss of *Cck*, *Gast*, *Gcg*, *Gip*, *Nts*, *Pyy*, and *Sct* expression in the duodenum (similar findings were noted in the colon) while the number of EC cells was unchanged compared to controls. The number of duodenal X/A and D cells were either unchanged or increased as progenitors are redirected to other lineages (Beucher et al., 2012; Du et al., 2012). In contrast, *Pax4* deficiency resulted in a reduction or complete loss of *Cck*, *Gast*, *Gip*, *Nts*, *Pyy*, *Sct*, *Sst*, and *Tph1* (a marker of EC cells) expression in the duodenum (similar findings were noted in the colon), but with an increase in GLP1-expressing cells (Beucher et al., 2012; Larsson et al., 1998). Interestingly, only one study showed that duodenal *Cck* and *Pyy* expression were also reduced in *Pax4*-deficient mice (Larsson et al., 1998), while another study suggested that *Pax4* deficiency increases colonic *Pyy* expression (Beucher et al., 2012).

These studies suggest that there are not clear-cut EE cell progenitor lineages that produce only 5-HT-secreting cells and peptide hormone-secreting cells, and that ARX and PAX4 do not strictly delineate two distinct lineage pathways, as some hormones require both transcription factors for production (notably GAST, GIP, NTS, and potentially CCK and PYY). Of note, ZNF800 deletion in human organoids increased the expression of the EC cell markers *TPH1* and *PAX4*, while multiple markers of peptide-hormone secreting cells were decreased, including *SST* and *ARX* (Lin et al., 2023). This suggests that ZNF800 suppresses *PAX4* expression, that ZNF800 is possibly expressed throughout the EE cell lineage, and that in human intestinal cells, PAX4 mainly specifies the EC lineage. The narrow role of PAX4 in human intestinal cells is supported by another study that added the small molecule isoxazole 9 (ISX-9), a known activator of NEUROD1, to human ileal organoids during differentiation (Tsakmaki et al., 2020). ISX-9 increased expression of *CCK*, *NEUROD1*, *PAX4*, and *TPH1* and decreased expression of *GCG*, *GHRL*, *PYY*, and *SST*, with the total number of EE and EC cells being increased compared to controls. This suggests that increased PAX4 is associated with 5-HT, but not SST, production in humans. It also suggests that NEUROD1 does not regulate the majority of peptide hormone expression in the ileum. Notably, similar results were found in mouse small intestinal organoids treated with ISX-9, except *Sst* and *Ghrl* levels were unchanged, and *Gip* levels were decreased. Interestingly, ISX-9 also increased the expression of *NEUROG3* in human organoids and increased the number of *Neurog3*-positive cells in mouse organoids, suggesting NEUROD1 may function upstream or with NEUROG3 (Tsakmaki et al., 2020). Another factor that may function throughout the EE cell lineage is Forkhead box O1 (FOXO1). Small molecule inhibition of FOXO1 in human duodenal organoids led to upregulation of multiple EE cell lineage markers, including *GIP*, *NEUROD1*, *NEUROG3*, *PAX4*, *PDX1*, and *SST*, increased the number of EC cells, and decreased *CCK* expression when compared to controls. FOXO1 inhibition also reduced the enterocyte marker *ALPI* and increased expression of *ATOH1*, suggesting it may play a role at the stem cell level (Zeve et al., 2022). Additionally, FOXO1 was also found to be a transcriptional target of insulin in a murine EE cell line, with insulin increasing dissociation of FOXO1 from the *Gip* promoter to induce increased *Gip* expression, suggesting it may stay involved in the later stages of the EE cell lineage (Garcia-Martinez et al., 2014).

All other transcription factors acting downstream of ARX/PAX4 to specify distinct lineages are summarized in Fig. 2B. These include paired box 6 (PAX6) and pancreatic and duodenal homeobox 1 (PDX1), which have also been implicated in EE cell differentiation of peptide-hormone secreting cells. *Pax6* knockout mice showed a reduction in *Cck*, *Gast*, *Gip*, and *Sst* expression while another study noted that PAX6 increased *Gcg* promoter activity in a murine intestinal cell line (Fujita et al., 2008; Larsson et al., 1998). One study using *Pdx1* knockout mice showed a significant reduction in duodenal *Gip* and *Sst* expression (Chen et al., 2009) while another study showed decreased numbers of duodenal GIP-, NTS-, SCT-, SST- and gastric GAST- and PYY-expressing cells (Larsson et al., 1996). An additional group of transcription factors that work primarily within the peptide-hormone secreting lineage are ISL LIM homeobox 1 (ISL1), and forkhead box protein A1 and A2 (FOXA1 and FOXA2). Intestinal ablation of *Isl1* resulted in the loss of CCK-, GIP-, GLP-1-, and SST- and an increase in 5-HT-producing cells (Terry et al., 2014), while *Foxa1* and *Foxa2* knockout mice showed decreased expression of small intestinal *Gcg*, *Pyy*, and *Sst*, along with significantly reduced levels of *Isl1* and *Pax6*, suggesting that FOXA1 and FOXA2 may act upstream of these transcription factors (Ye and Kaestner, 2009).

Several transcription factors have been identified exclusively within the EC cell lineage. Lim homeobox transcription factor 1 alpha (LMX1A) and ETS family member FEV are expressed in 5-HT-producing cells, functioning downstream of NKX2–2. Deletion of *Lmx1a* reduced the number of murine EC cells, suggesting it is necessary for intestinal 5-HT production (Gross et al., 2016), but a recent study deleting LMX1A in human intestinal organoids did not show any change in EC cell number (Beumer et al., 2020). And, although a recent murine single cell analysis showed *Fev* expression very early in EC development (Gehart et al., 2019), *Fev* deletion did not alter EE cell or EC cell number in mice, suggesting that its role in EC cells is redundant (Wang et al., 2010). Interestingly, LMX1A was originally noted to be specific for EC cells while its homologue LMX1B was shown to be specific for 5-HT-secreting neurons. However, a recent transcriptomic study noted LMX1B expression in the EE cell lineage, suggesting both LMX1A and LMX1B possibly play a role in EC cell differentiation (Singh et al., 2024). Additionally, *Glis3* was found to be enriched in EC cells in a single cell RNA-sequencing study in mouse organoids (Piccand et al., 2019).

Another transcription factor that may function throughout the EE cell lineage is regulatory factor X6 (RFX6). Knocking out *Rfx6* in adult mouse intestine induced decreased expression of all peptide hormones and multiple peptide-producing EE cell markers, including *Arx*, *Isl1*, and *Pax6*. Further, EC-specific transcription factors, like *Pax4* and *Lmx1a*, were upregulated. Interestingly, organoids derived from these *Rfx6* knockout mice had decreased expression of the EC markers *Tph1* and *Lmx1a*, suggesting a possible difference between *in vivo* and *ex vivo* EE cell differentiation. Further, this same study found that deletion of *Rfx6* upregulated *Neurod1*, *Neurog3* and *Nkx2-2*, while *Sox4* and *Atoh1* expression levels were unchanged (Piccand et al., 2019). A recent study using human tissue and iPSC-derived organoids from a patient with an *RFX6* variant found decreased expression of *CHGA* and *PDX1*, as well as a decrease in CHGA-positive cells, suggesting that PDX1 acts downstream of RFX6 (Sanchez et al., 2024). Insulinoma-associated 1 (INSM1) was also similarly found to play a broad role throughout the EE cell lineage. Knocking out *Insm1* in mice led to a significant reduction in CCK-, NTS-, 5-HT-, and PYY-positive cells, but did not significantly alter the number of SCT-positive cells. Further, *Insm1* knockout had no effect on expression of *Neurod1* and *Neurog3* (Gierl et al., 2006), suggesting INSM1 functions downstream of these factors. To support this, it has been noted that overexpression of *Neurod1* in murine intestinal tissue increased expression of *Insm1* (Li et al., 2019a). Inhibitor of DNA binding 2 (ID2) has also been suggested to play a broad and evolving role throughout EE cell differentiation. In *Id2* conditional knockout within the adult intestine, total EE cell numbers were decreased while the number of tuft cells increased in the small intestine, suggesting ID2 may first act at the secretory progenitor level to limit tuft cell differentiation. In these knockout mice, ileal PYY- and NTS-producing cell numbers were reduced, EC cells and GHRL-positive cell numbers were increased, and CCK-, GLP-1-, and SST-producing cell numbers were unchanged. This suggests that ID2 also acts later within the EE cell lineage to block differentiation of X/A and EC cells in the ileum and to mediate differentiation of PYY- and NTS-positive cells (Zinina et al., 2022).

One recent study combined loss-of-function models with single cell RNA sequencing and lineage tracing to discover several novel transcription factors in murine EE cell differentiation, as well as to validate previous findings (Gehart et al., 2019). Using a fluorescent reporter of NEUROG3 that tracks age and lineage of EE cells from the onset of *Neurog3* expression, EE cells were isolated at various stages and transcriptional regulators were identified at each stage using single cell sequencing. A subsequent CRISPR screen in murine intestinal organoids identified six regulators that specify different EE cell phenotypes: SOX4, RFX6, Tox high mobility group family member (TOX3), myelin transcription factor 1 (MYT1), RUNX1 Translocation Partner 1 (RUNX1T1), and Zinc finger CCHC-type containing 12 (ZCCHC12) (Gehart et al., 2019). The screen confirmed SOX4 plays a broad but essential role in driving EE cell differentiation. Additionally, *Rfx6* deletion induced decreased expression of *Cck*, *Gip*, *Gcg*, *Ghrl*, and *Tph1*, similar to the other Rfx6 knockout study in organoids, though, as noted above, these results differ from some found *in vivo* (Piccand et al., 2019). *Tox3* and M*yt1* deletion decreased *Tph1* expression, suggesting they are involved in EC cell lineage differentiation; however, *Tox3* deletion also increased *Ghrl* expression, suggesting that it may function earlier in the EE cell lineage. Loss of *Zcchc12* resulted in decreased *Ghrl* expression, and loss of *Runx1t1* resulted in decreased expression of *Cck*, *Gip*, and *Sst* (Gehart et al., 2019). The study also found several transcription factors that were not pursued via CRISPR but seemed to regulate distinct lineages: One cut homeobox 3 (ONECUT3) was enriched in I and N cells, poly (*ADP-ribose*) polymerase 1 (PARP1) was enriched in D, L and EC cells, and ETS variant 1 (ETV1) was enriched in I, L, and N cells (Gehart et al., 2019). Interestingly, in another study, *Etv1* was noted to be expressed significantly higher in small intestinal L cells when compared to K cells, along with prospero homeobox 1(PROX1) (Habib et al., 2012). PROX1 appears to have a broad role in peptide-hormone producing EE cells, as it has been shown to be co-expressed with CCK, GLP1, and PYY in murine small intestine (Petrova et al., 2008). Additionally, a study of mouse jejunum showed that *Prox1* was co-expressed with *Cck*, *Ghrl*, *Gip*, *Pyy*, and *Sct*, as well as with several tuft cell markers, suggesting a role for PROX1 in multiple secretory lineages (Yan et al., 2017). Finally, High Mobility Group Nucleosomal Binding Domain 3 (HMGN3) and PR/SET Domain 16 (PRDM16) were found to be highly expressed in EC cells, along with *Fev* (Gehart et al., 2019); however, it is unclear what role, if any, HMGN3 and PRDM16 play in EE cell differentiation. It is notable that postnatal deletion of *Prdm16* leads to an increased number of EE and goblet cells in the duodenum, suggesting PRDM16 might function early in ISC differentiation (Stine et al., 2019).

Other transcription factors have been identified that may drive differentiation of specific EE cell lineages. For example, suppression of GATA binding protein 4 (*Gata4)* via RNAi resulted in reduced *Gip* transcription and GIP secretion in multiple murine cell lines, while overexpression enhanced *Gip* transcription and GIP secretion, suggesting GATA4 plays a role in K cell differentiation (Jepeal et al., 2008). Hepatocyte nuclear factor 4α (HNF4α) has also been shown to regulate incretin hormones; *Hnf4α* knockout mice had reduced *Gip* and *Gcg* expression along with decreased tissue levels of jejunal GIP and colonic GLP1 when compared to controls. Additionally, although there was a significant decrease in the number of colonic GLP1-positive cells, colonic GIP-positive cells were unchanged compared to controls (Girard et al., 2019). Peroxisome proliferator-activated receptor beta and delta (PPARβ and PPARδ) activation was also shown to increase *Gcg* expression and GLP1 release in human and murine L cells (Daoudi et al., 2011). Further, haematopoietically expressed homeobox protein (HHEX) has been implicated in formation of D cells, as deletion of *HHEX* in human intestinal organoids decreased the number of SST-positive cells (Beumer et al., 2020). Excitingly, multiple recent, large scale transcriptomic and screening studies have identified many other transcription factors that could regulate the EE cell lineage, allowing the field to continue to study this complicated process for years to come (Beumer et al., 2020; Gebert et al., 2020; Singh et al., 2024).

As our understanding of the transcriptional regulators of EE cell differentiation grows, it becomes evident that this process is not straightforward. The production of multiple hormones within a single EE cell suggests that these transcription factors most likely work in flux or in parallel, with dynamic expression of transcription factors, and eventually hormones, being driven by external cues, including metabolic states and GI function. Although this has not been proven, the ability of EE cells to dedifferentiate into ISCs in the setting of injury (Yan et al., 2017) suggests that these cells are not static and may be able to alter their differentiated state to produce different hormones as needed.

## 4. Signaling pathways regulating enteroendocrine cell differentiation

**Fig. 3.**
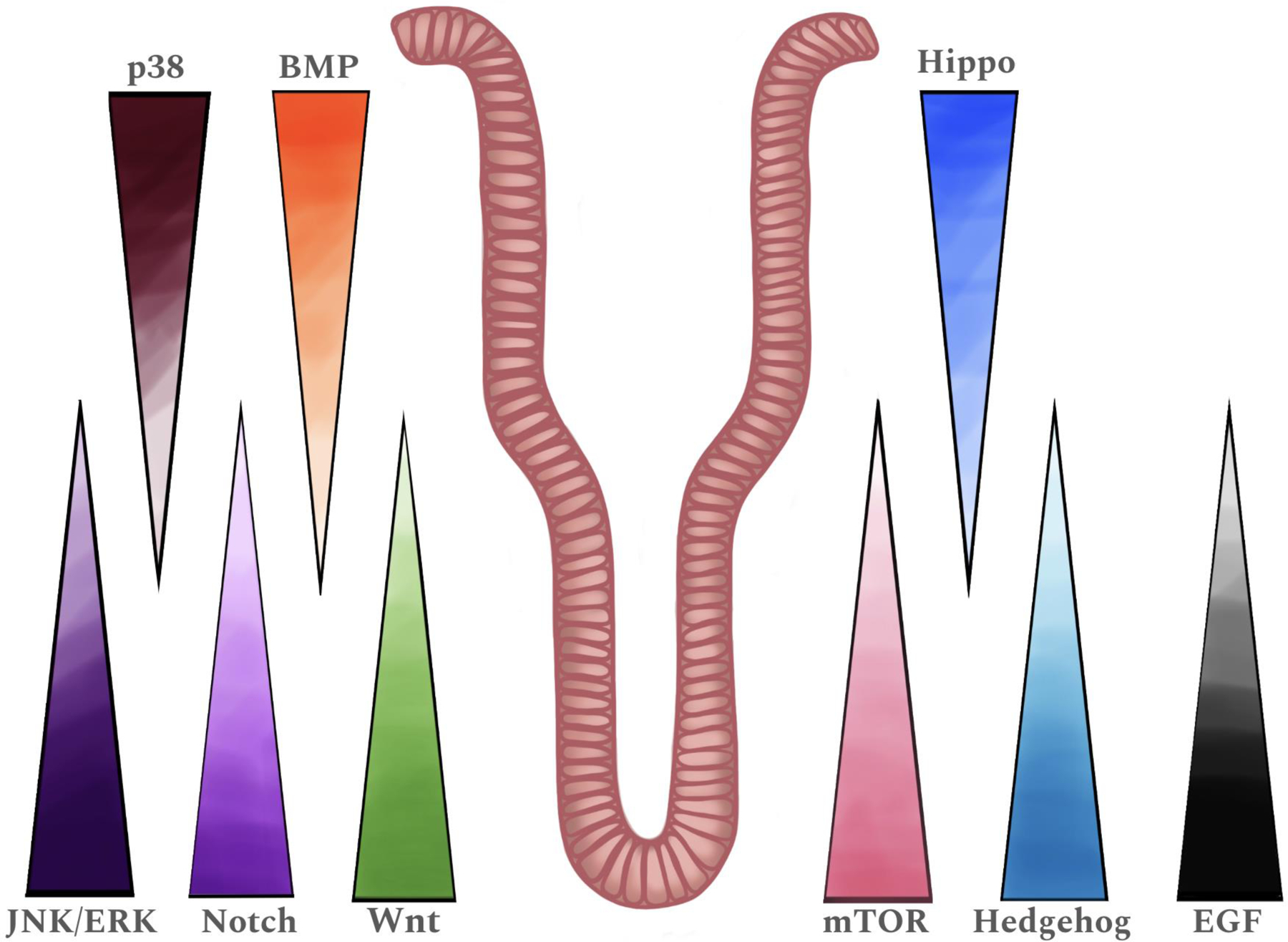
Signaling Pathway Activity in the Crypt-Villus Axis The signaling pathways governing intestinal stem cell differentiation exhibit a gradient of activity along the crypt-villus axis, with differential expression of receptors and ligands either in the crypts (stem cell compartment) or the villi (mature cells), contingent upon the specific pathway involved. Notably, the endocannabinoid signaling pathway is difficult to characterize as there are receptors in both the crypts and the villi; cannabinoid receptor type 1 (CNR1) is expressed in the crypt, as well as in goblet and absorptive cells of the villi, while CNR2 is predominantly expressed in Paneth cells.

The development and maintenance of EE cells are modulated by several signaling pathways that act in concert throughout the EE lineage to dictate the production and function of these cells (reviewed in Beumer and Clevers, 2021; Kolev and Kaestner, 2023; Takahashi and Shiraishi, 2020). Many of these signaling pathways have been thoroughly studied and reviewed in terms of EE cell differentiation, including the Wnt signaling and Notch signaling pathways, among others. For this review, we will give a brief overview of specific signaling pathways, how they regulate EE cell differentiation, and recent studies that update their roles. Further, we will focus on less studied pathways that may also play a role in EE cell differentiation. The signaling pathways discussed, their roles in promoting or inhibiting differentiation and their activity in the crypt-villus axis are summarized in Table 1 and Fig. 3, respectively.

The Wnt signaling pathway is well known to regulate ISC function, modulating cellular proliferation and differentiation, and has been thoroughly studied and reviewed. The canonical Wnt pathway involves a Wnt ligand binding to a Frizzled receptor and LRP5/6 co-receptor, leading to stabilization and nuclear translocation of β-catenin, which acts as a transcriptional co-activator to regulate gene expression. Active Wnt signaling is crucial for maintenance of ISC function and inhibition of ISC differentiation. Previous studies have shown that the crypt-villus axis has a decreasing gradient of Wnt signaling, which is used to maintain stemness within the crypt and to promote intestinal epithelial cell differentiation as cells move up to the villus (Beumer and Clevers, 2021; Flanagan et al., 2018; Koch, 2017; Mah et al., 2016). In fact, multiple studies have shown that the removal of Wnt ligands (typically WNT3A-containing conditioned media) or the addition of Wnt signaling inhibitors are necessary for *in vitro* ISC differentiation (reviewed in Beumer et al., 2018; Pleguezuelos-Manzano et al., 2020; Sato et al., 2011; Wilson et al., 2020; Yin et al., 2014). However, other studies have suggested that reduction, but not complete removal, of Wnt ligands is not detrimental to human ISC differentiation (Dieterich et al., 2020; Mohammadi et al., 2021; Tsakmaki et al., 2020; VanDussen et al., 2015). Moreover, additional studies showed that the level of WNT3A-containing conditioned media can be maintained at the level required to sustain human ISCs while promoting EE cell differentiation (Fujii et al., 2018; Zeve et al., 2022). It has also been suggested that Wnt signaling may need to be present for longer than previously believed in murine organoids to drive EE cell differentiation. Using the Wnt-activator CHIR, it was noted that maintaining Wnt signaling for an additional day at the start of a differentiation protocol was able to increase EE cell numbers when compared to inhibiting Wnt signaling during that time. Notably, this five-day differentiation protocol inhibited Wnt signaling during its final three days (Yang et al., 2024).

The non-canonical Wnt signaling pathway, in which specific Wnt ligands, distinct from those involved in the canonical Wnt pathway, induce calcium or planar cell polarity (PCP) signaling to activate gene expression independent of β-catenin (Qin et al., 2024), has also been shown to play a role in EE cell differentiation. A recent study in mice has shown that activation of non-canonical Wnt/PCP signaling drives ISCs into both EE cell and Paneth cell lineages. Using multiple mouse models and single cell RNA sequencing, it was shown that non-canonical Wnt/PCP signaling in ISCs leads to directed differentiation of either EE cells or Paneth cells without going through a common secretory progenitor cell intermediate. Interestingly, ISCs experiencing either canonical or non-canonical Wnt/PCP activation showed similar transcriptional profiles, suggesting that the switch from canonical to non-canonical Wnt/PCP signaling may involve post-transcriptional modifications (Bottcher et al., 2021).

Another well studied pathway that plays a pivotal role in EE cell differentiation is the Notch signaling pathway, which works through Notch ligands binding to the Notch receptor, triggering a series of proteolytic cleavage events leading to the release of the Notch intracellular domain (NICD). The NICD translocates to the nucleus, where it interacts with transcription factors to regulate the expression of target genes involved in cell differentiation, proliferation, and apoptosis. As described previously, high levels of Notch signaling promotes ISC differentiation into the absorptive lineage through increased *HES1* expression, while low/inhibited Notch signaling promotes secretory lineage differentiation (Demitrack and Samuelson, 2016), which explains why many intestinal organoid EE cell differentiation protocols contain a Notch signaling inhibitor (Yang et al., 2024; Zeve et al., 2022). Interestingly, even though inhibition of Notch signaling is required for EE cell differentiation, it has been shown that EE cells themselves are able to promote Notch signaling in surrounding cells, aiding in maintaining ISC identity. This was first described in Drosophila (Guo and Ohlstein, 2015), which have a simple (by comparison) intestinal epithelium consisting of stem/progenitor cells, absorptive cells, and EE cells, and was later proven true in mammals. In a mouse model lacking Paneth cells, which typically drive Notch signaling in ISCs, EE cells and tuft cells were shown to migrate to the stem cell niche and activate ISC Notch signaling through expression of Notch ligands, helping to maintain ISC function (van Es et al., 2019).

Like Wnt signaling, the BMP signaling pathway also involves spatially guided differentiation along the crypt-villus axis (Beumer et al., 2018, 2022), although in contrast to Wnt signaling, BMP signaling increases with differentiation. Canonical BMP signaling, which functions through SMAD transcription factors (Zhang and Que, 2020), has been shown to drive general ISC differentiation and the eventual depletion of all ISCs. Because of this, protocols designed for intestinal organoid culture typically contain Noggin, an antagonist of BMP signaling (Pleguezuelos-Manzano et al., 2020; Wilson et al., 2020; Zeve et al., 2022). Within the EE cell lineage, it has been shown, in both mouse and human, that BMP signaling can alter specific EE hormone expression. For example, murine intestinal organoids treated with varying time-courses of BMP4, mimicking the exposure to BMP along the crypt-villus axis, induced altered hormone expression in K cells (among others). With only one day of BMP4 exposure, K cells expressed high levels of *Gip* and *Cck*, but with four days of BMP4 exposure, *Sct* and *Pyy* were also expressed (Beumer et al., 2018). BMP activation can also affect other intestinal cell types; a recent study in human intestinal organoids showed that BMP signaling can alter gene expression in both enterocytes and goblet cells (Beumer et al., 2022). Interestingly, a study in mouse intestinal tissue showed that inhibiting BMP signaling through deletion of the BMP receptor *Bmpr1a* increased the total number of EE cells within the stomach, with increased numbers of D, EC, G, and X/A cells when compared to controls, but decreased the total number of EE cells within the duodenum (Maloum et al., 2011). This suggests that BMP signaling through BMPR1A may function differently to mediate ISC differentiation depending on GI tract location.

EGF signaling has also been shown to regulate ISC differentiation. This signaling pathway relies on the interaction of multiple ligands (including EGF, amphiregulin, neuregulin (NRG), epiregulin (EREG), and betacellulin (BTC), amongst others), with four members of the Erb family of tyrosine kinase receptors (EGFR (ErbB1), ErbB2, ErbB3, and ErbB4) (Abud et al., 2021). EGF, NRG, and EREG are generally expressed in the crypts, with some expression found in mesenchymal cell populations (Abud et al., 2021; Childs et al., 2023; Jarde et al., 2020; Liu et al., 2017). Through combinations of ligand-receptor binding and receptor heterodimerization, EGF signaling activates multiple downstream factors to regulate transcription. In the intestine, EGF signaling is associated with ISC stemness (Abud et al., 2021). In organoid culture, it has been shown that removal of EGF during differentiation improves overall mature cell development (Fujii et al., 2018). Interestingly, a study in murine organoids showed that the use of gefitinib, an EGFR inhibitor, during ISC differentiation did not alter EE cell numbers, but the later removal of gefitinib during differentiation, with or without the reintroduction of EGF, increased the number of CHGA-positive cells (Basak et al., 2017). Other studies have utilized continued exposure to EGF without inhibition of EE cell differentiation and shown that the addition of BTC improves EE cell differentiation (based on *CHGA* expression) (Shepherd et al., 2024; Zeve et al., 2022).

NRG1 has also been implicated in EE cell differentiation. Compared to human fetal duodenal organoids grown in EGF, those grown in NRG1 were able to establish multiple mature cell types, including a small number of EE cells and goblet cells (Holloway et al., 2021). Similarly, it was noted that EREG acted in a similar fashion to NRG1, inducing EE cell differentiation in human fetal duodenal organoids. Interestingly, the largest increase in *CHGA* expression was seen after organoids were grown in EGF for two passages and then switched to EREG (Childs et al., 2023). Since EGF, EREG, and NRG1 have different receptor specificity (EGF binds to EGFR, EREG to EGFR and Erbb4, and NRG1 to Erbb3 and Erbb4) (Abud et al., 2021), it is possible that activation of different Erb family heterodimers may impact the balance between stem, secretory, and absorptive lineages.

The MAPK signaling pathway is also well-known to regulate EE cell differentiation. This pathway responds to multiple growth factors, cytokines, and stressors to signal through three main targets, JNK, p38, and ERK, all of which alter transcription to regulate cell survival and differentiation (Braicu et al., 2019; Cargnello and Roux, 2011; Zhang and Liu, 2002). In mouse models, p38 has been shown to be active in primarily the mature cells of the villus, suggesting a positive role in differentiation (Houde et al., 2001). In support of this, during *in vitro* culture of human intestinal organoids, protocols designed for ISC maintenance usually include a p38 inhibitor to prevent differentiation (Pleguezuelos-Manzano et al., 2020; Zeve et al., 2022). While p38 has been shown to promote ISC differentiation, both ERK and JNK have the opposite effect (Cui and Chang, 2016; Lemieux et al., 2011; Osaki and Gama, 2013; Semba et al., 2020). It has been shown that ERK signaling is mostly active in the crypts, with little to no activation in the villi (Lemieux et al., 2011; Pond et al., 2022); however, one murine study suggests that ERK signaling increases along the crypt-villus axis (Kabiri et al., 2018). In support of ERK maintaining stemness, previous studies have shown that ERK inhibitors can strongly induce EE cell differentiation in mouse and human intestinal organoids (Pleguezuelos-Manzano et al., 2020). JNK signaling, based on phosphorylation of the c-Jun protein, has been identified in crypts, although the c-Jun protein itself is found in both the crypt and villus (Hasselblatt et al., 2008; Sancho et al., 2009). Consistent with this, the addition of a JNK inhibitor to human intestinal organoids also induced EE cell differentiation, but to a smaller extent (Zeve et al., 2022). These studies, along with many others, show that JNK and ERK signaling serve to maintain the ISC state and inhibit secretory differentiation, while p38 MAPK promotes ISC differentiation.

Often acting alongside MAPK signaling, the mTOR pathway, working through mTOR complex 1 and 2 (mTORC1 and mTORC2), also signals downstream of multiple stimuli, including nutrients, stress, and growth factors, to regulate a myriad of cellular functions (Panwar et al., 2023; Saxton and Sabatini, 2017). The mTOR pathway is believed to exhibit the highest level of activity in the crypt, with a gradual decrease in activity along the crypt-villus axis (Fujishita et al., 2008; Yang et al., 2016). In intestinal tissue, multiple studies reveal that mTORC1 functions to inhibit EE cell differentiation. Knocking down a component of mTORC1 (*Raptor*) using RNAi in Drosophila significantly increased the number of EE cells in the fed state (Mattila et al., 2024). Murine studies have also evaluated the role of mTORC1 in ISC differentiation. One study showed that *Raptor* deletion in adult mice increased the number of EE cells within the small intestine, while mTORC1 activation had no effect on EE cell numbers compared to controls (Barron et al., 2017). Another study, focusing on the ileum, showed that deletion of *mTOR* (found in both mTORC1 and mTORC2) led to increased EE cell differentiation, while goblet cell numbers were unchanged. Although Paneth cells were not counted, decreased expression of the Paneth cell marker *Lyz* was also noted. This effect on the EE cell lineage appeared to be secondary to mTORC1 only, as *Rictor* (specific for mTORC2) knockout mice did not show altered EE cell differentiation (Sampson et al., 2016). Despite the above data, addition of an mTOR inhibitor to mouse intestinal organoids inhibited EE differentiation, even in the setting of Wnt, Notch, and EGFR inhibition (Basak et al., 2017).

mTOR signaling also regulates hormone production and secretion. Compared to controls, mice treated with the mTOR inhibitor rapamycin had decreased levels of *Gast* mRNA and circulating plasma GAST levels, increased levels of *Ghrl* mRNA and circulating total ghrelin levels, and unchanged levels of *Sst* mRNA and circulating SST levels (Xu et al., 2010). Further defining the role of mTOR in ghrelin production, specific activation of mTORC1 in X/A cells decreased *Ghrl* mRNA levels and plasma levels of both acylated and total GHRL without altering the number of X/A cells when compared to controls (Yu et al., 2018). Along with this, specific deletion of *mTOR* in X/A cells increased plasma acylated GHRL levels (Li et al., 2019b). Outside of ghrelin, it was noted that inhibition of mTORC1 through multiple methods, but not mTORC2, in a human endocrine cell line induced increased *NTS* gene expression (Li et al., 2011), while rapamycin administration led to a decrease in ileal *Gcg* expression and plasma GLP1 levels in mice (Xu et al., 2015). Conversely, activation of mTOR signaling, either through administration of amino acids or genetically by targeting *Neurog3*-expressing cells, induced increased ileal *Gcg* expression and plasma GLP1 levels (Xu et al., 2015). Overall, it appears that mTOR signaling through mTORC1 decreases EE cell differentiation, while its role in hormone production appears hormone specific.

Other major pathways known to regulate stem cell function have not been extensively evaluated or have been shown to have little or no role in EE cell differentiation. This includes the Hedgehog signaling pathway, which regulates intestinal development and involves the binding of Hedgehog ligands (such as Sonic Hedgehog (SHH), Indian Hedgehog (IHH), and Desert Hedgehog (DHH)) to the Patched receptor, allowing the Smoothened receptor to activate downstream GLI transcription factors (Carballo et al., 2018; Walton and Gumucio, 2021). It has been suggested that intestinal expression of SHH and IHH is predominantly localized to the crypts rather than the villi, further suggesting their potential involvement in ISC regeneration via paracrine signaling (Walton and Gumucio, 2021). Multiple mouse models have been produced to examine ligand-specific knockouts in murine intestine. IHH is the major hedgehog ligand expressed in the small and large intestine and one study showed that intestine-specific deletion led to an increase in multiple secretory lineages, including EE cells, with increased expression of *Gcg* and *Cck* (Kosinski et al., 2010). However, other studies have shown no change in EE cell numbers in *Ihh* knockout mice compared with controls (van Dop et al., 2010; Zacharias et al., 2011). Further, the EE cell lineage is not altered in the setting of either *Shh* deletion or over-expression (Gagne-Sansfacon et al., 2014; Liang et al., 2012; Zacharias et al., 2011). Of note, inhibition of Hedgehog signaling using small molecules did not alter EE cell marker expression in murine organoids (Beumer et al., 2018).

Hippo signaling, another evolutionarily conserved pathway regulating stem cell function, signals through multiple kinases to inhibit the co-activators YAP/TAZ from driving transcription, typically through the TEAD transcription factors (Fu et al., 2022; Hong et al., 2016). Several studies have shown that YAP activity decreases along the crypt-villus axis and that YAP regulates expression of the ISC marker Lgr5. As YAP is inversely associated with Hippo pathway activation, the Hippo pathway is active in the villi while mostly inactive in the crypts (Deng et al., 2022; Tsutsumi et al., 2013). In flies and in mouse organoids, inhibition of Hippo signaling did not alter EE cell differentiation (Aihara et al., 2022; Karpowicz et al., 2010; Shaw et al., 2010). In a human intestinal cell line, knockdown of *YAP* also did not change expression of *CHGA* but did increase expression of *ATOH1* and *MUC2* (Fallah and Beaulieu, 2020). In contrast, double knockdown of *Yap/Taz* in mice showed decreased numbers of goblet cells and *Atoh1* expression, but no change to EE cells (Imajo et al., 2015). Based on these data, it is unclear how and if Hippo signaling regulates secretory lineage differentiation, but it is notable that a transcriptomic study of EE cell differentiation showed TEAD transcription factors as being expressed early during lineage specification (Gehart et al., 2019), suggesting a positive role in secretory differentiation.

Finally, the endocannabinoid pathway, which signals through cannabinoid receptor type 1 and 2 (CNR1 and CNR2), has also been shown to regulate EE cell lineage differentiation and EE hormone production, specifically related to appetite regulation (DiPatrizio, 2016, 2021). CNR1 is found to be expressed in the crypt as well as on Goblet and absorptive cells, while the CNR2 receptor is found mainly on Paneth cells (Cherkasova et al., 2021). One study evaluated the secretion of GLP1 and GIP in adult patients given a CNR agonist and found that it increased both fasting and postprandial levels of GIP but decreased postprandial levels of GLP1 (Chia et al., 2017). In addition, treatment of mice with a CNR1 agonist inhibited fat-induced secretion of CCK (Argueta et al., 2019). Further, patients with polycystic ovary syndrome (PCOS) treated with rimonabant, a CNR1 inverse agonist, were found to have increased plasma levels of GIP after three months, but GLP1 levels were not changed (Sathyapalan et al., 2010). In a mouse ghrelinoma cell line, exposure to a CNR1 antagonist decreased the production of acylated ghrelin (Godlewski et al., 2019). Finally, a study using human duodenal organoids showed that exposure to rimonabant during ISC differentiation induced expression of *CHGA*, *NEUROD1*, *NEUROG3*, *SST*, and *GIP* (Zeve et al., 2022). Moreover, when rimonabant was paired with JNK inhibition, *ATOH1*, *MUC2*, and *LYZ* expression were also increased. Finally, human organoids treated with rimonabant and JNK inhibition showed increased production of multiple hormones, including SST, 5HT, CCK and GIP, with CCK and GIP being more highly produced compared to other EE cell differentiation protocols (Zeve et al., 2022). These data suggest that endocannabinoid signaling inhibits EE cell differentiation, inhibits the production of anorexigenic hormones, and promotes production of orexigenic hormones.

EE cell differentiation is a complex process regulated by a network of transcription factors and pathways. Our ever-growing understanding of EE cell differentiation has provided a strong foundation for studying how aspects of human physiology and pathophysiology can alter this lineage, and how these changes can alter human health. Specifically, we will next examine EE cell differentiation and function in the context of aging, obesity, and inflammatory bowel disease (IBD), highlighting how altered EE hormone production can contribute to different pathological states.

## 5. Enteroendocrine cells and aging

**Fig. 4.**
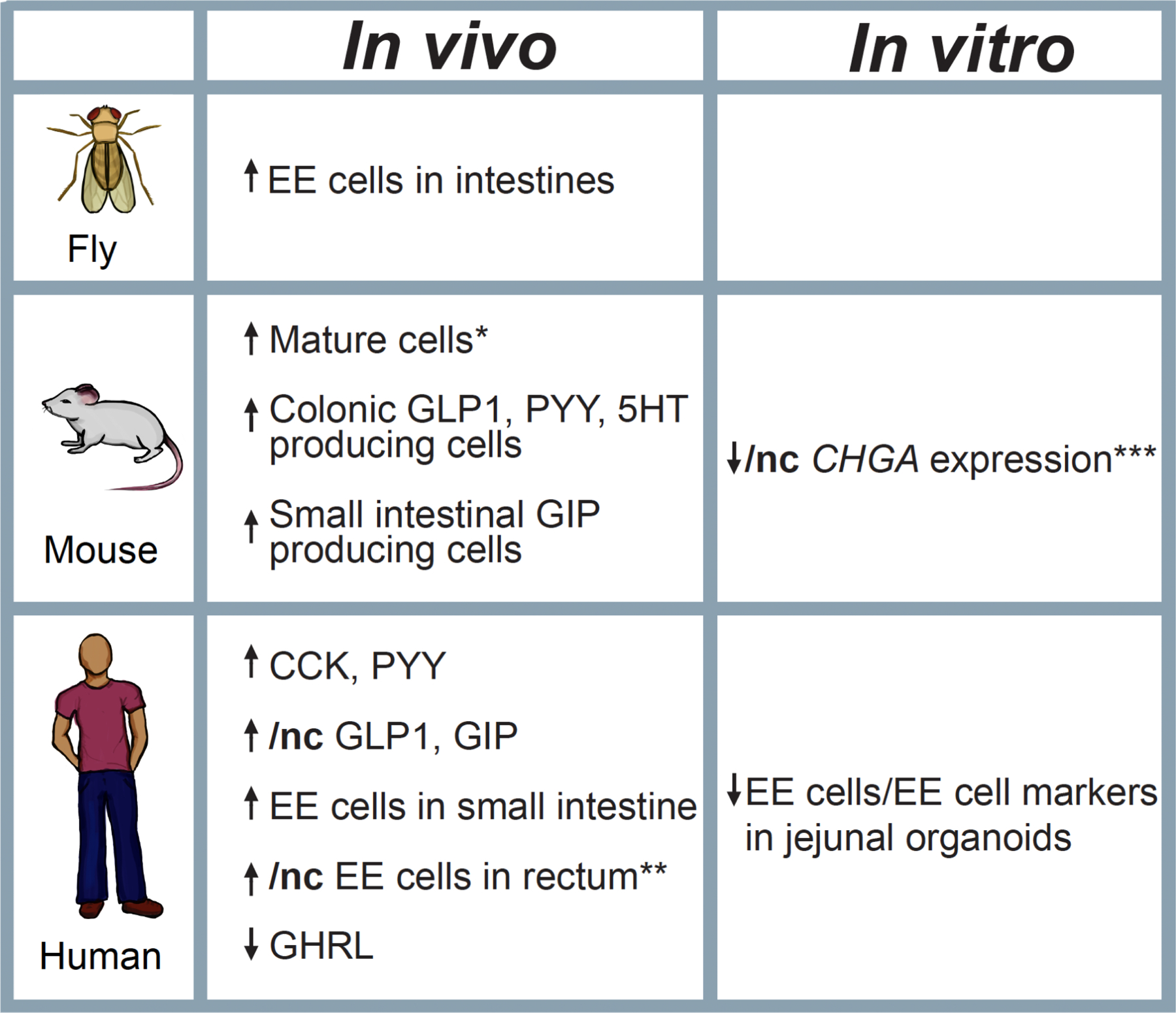
Age-induced Changes in Enteroendocrine Cell Number and Function Differences in EE cell number and hormone production in aged *in vivo* vs *in vitro* models. Mature cells = EE cells, tufts cells, goblet cells, Paneth cells, enterocytes. nc = no change * Two studies find no change in the number of crypt EE cells ** Only one study finds an increase in EE cells in the sigmoid colon and rectum, while others find no difference in rectal EE cell number with age. *** One study finds decreased CHGA expression, while another finds more EE progenitor cells/ISCs but no difference in CHGA expression.

**Fig. 5.**
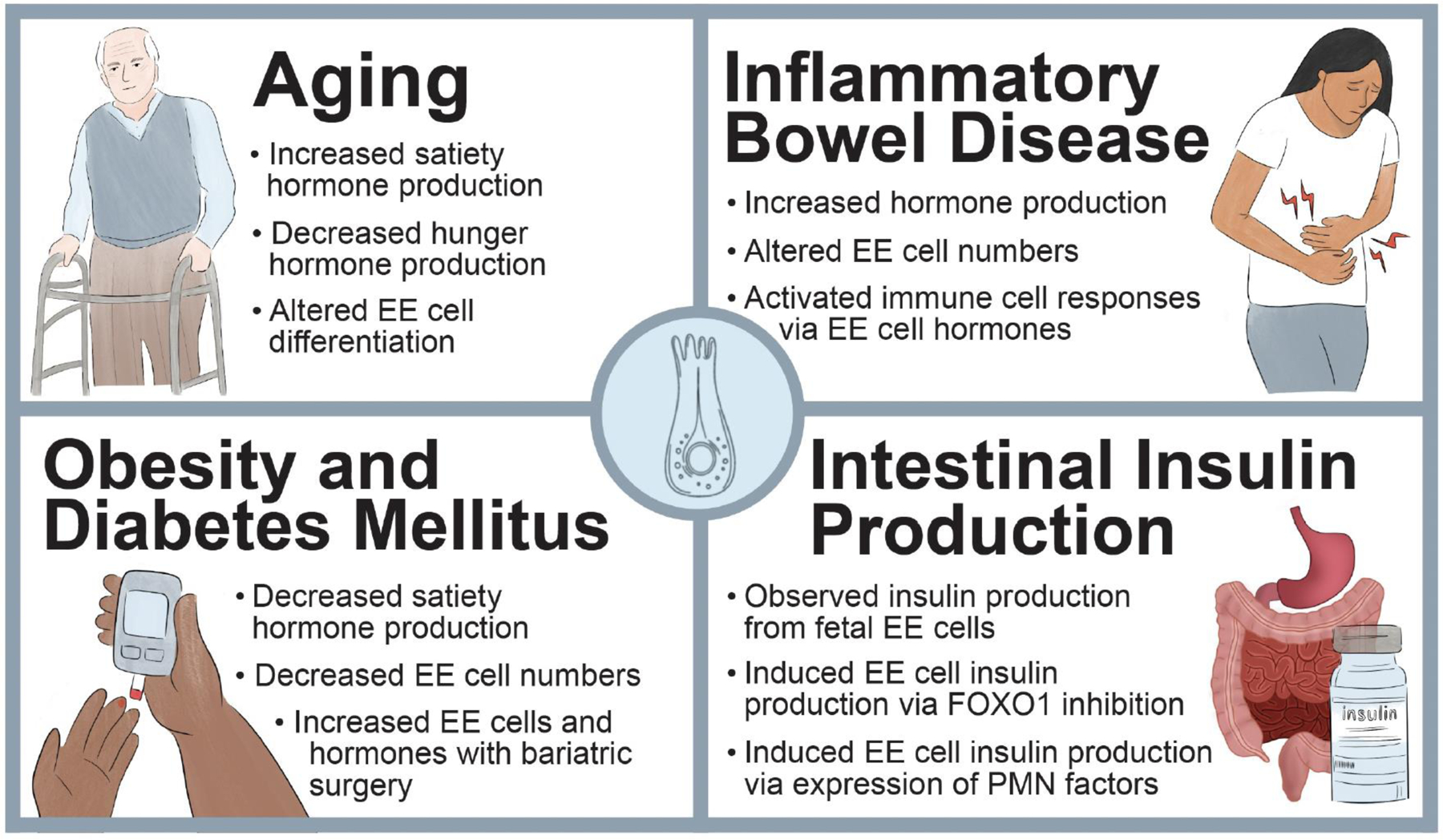
Enteroendocrine Differentiation and Human Health Changes in enteroendocrine (EE) cell differentiation and hormone production are involved in aging, IBD, obesity, and diabetes mellitus. Targeting EE cells to produce insulin is a potential therapy for diabetes mellitus. PMN = PDX1, MAFA, and NEUROG3.

Aging is associated with a myriad of changes within the GI tract. Once a person reaches 65 years of age, the incidence and severity of several disorders associated with GI motility (including dysphagia, gastroesophageal reflux, small intestinal bacterial overgrowth) increase and may result in a significant decline in quality of life due to malabsorption, diarrhea, and constipation (Dumic et al., 2019; Firth and Prather, 2002; Saffrey, 2014). Along with these, many older adults also suffer from decreased appetite, referred to as the anorexia of aging, which affects up to 30 % of community dwelling older adults and up to 85 % of those living in nursing homes, leading to increased wasting, frailty, and mortality (de Souto Barreto et al., 2022; Malafarina et al., 2013; Merchant et al., 2022; Wysokinski et al., 2015). Although the social and economic burden of the anorexia of aging has yet to be quantified, we know that frailty can increase healthcare costs almost three-fold when compared to robust older adults (Chi et al., 2021; Ensrud et al., 2020; Hoogendijk et al., 2019). While there are multiple social, economic, medical, and physiological components that drive the anorexia of aging, this review will focus on changes within the EE system, summarized in Figs. 4 and 5 (Malafarina et al., 2013; Merchant et al., 2022; Moss et al., 2012; Wysokinski et al., 2015).

Several studies have found altered production and/or response to EE cell hormones with age, all of which led to reduced appetite, including GHRL, CCK, PYY, and GLP1. However, due to most experiments being performed in humans, some of the data are contradictory. These previous studies have been reviewed extensively (Malafarina et al., 2013; Merchant et al., 2022; Moss et al., 2012; Wysokinski et al., 2015), so we will focus more on the overall changes seen with these hormones, highlighting more recent studies if possible. It has been suggested that GHRL secretion, and response, decreases with age; however, due to older studies being unable to differentiate between acylated and unacylated forms, it is difficult to interpret these changes in GHRL. A recent meta-analysis suggested that there was a small decrease in post-prandial acylated ghrelin in older adults (with the caveat being only one study actually fit their inclusion criteria) while there was little difference in total ghrelin levels between the fasted and postprandial state (Johnson et al., 2020). Recently, one study evaluated fasted and post-prandial ghrelin levels in 14 young adults (18–29 years) and 28 older adults (greater than 65 years). The older adults were further split into those with low appetite and high appetite. Interestingly, both fasted acylated and total ghrelin levels were increased with low appetite in older adults when compared to the other two groups, suggesting possible ghrelin resistance. Postprandial levels of acylated and total ghrelin levels were lower in both the older adult groups when compared to young adults, with the low appetite group showing a larger decrease, suggesting an exaggerated negative response from X/A cells after eating (Holliday et al., 2024).

CCK has been one of the more highly studied EE cell hormones in the setting of aging, with previous studies showing elevated fasted and postprandial levels of CCK in older adults, which was supported by a recent meta-analysis (Johnson et al., 2020). For example, a human study showed higher baseline plasma levels of CCK in older (67–83 years) compared to younger (18–33 years) subjects at baseline, and infusion of CCK (using weight-based dosing) suppressed food intake in older adults twice as much as in younger adults (MacIntosh et al., 2001). PYY is also believed to play a role in the anorexia of aging. Although one recent study suggested that PYY is unchanged in fasted older adults (Dagbasi et al., 2024), meta-analysis data showed PYY levels are actually lower when compared to younger adults (Johnson et al., 2020). One recent study analyzed different areas of the intestine and noted a negative relationship between basal GLP1 and PYY tissue levels within the colon and age (decreasing from about 40 to 80 years of age) (Jones et al., 2023). Postprandially, most studies agree that PYY is increased in older adults when compared to younger adults (Johnson et al., 2020), leading to early satiety. However, one recent study suggested that postprandial PYY levels are not significantly different in older compared to younger adults, although a trend for increased PYY in older adults was present (Dagbasi et al., 2024).

Changes in GIP and GLP1 with age are not as obvious as CCK, GHRL, and PYY. Meta-analysis data suggested that there are no fasting or postprandial changes in GLP1 with age; however, there are small increases in fasting and postprandial levels of GIP (Johnson et al., 2020). A different meta-analysis focusing on GIP secretion in T2DM showed that patients younger than 60 years of age had increased GIP secretion compared to those older than 60 years, although other variables, including glycemic control and time since T2DM diagnosis, could possibly alter EE hormone secretion (Calanna et al., 2013). One study analyzed GIP and GLP1 levels in older adults by performing a longitudinal study in 41 healthy patients 67–75 years. Fasted GLP1 and GIP levels were drawn, and a glucose tolerance test was performed. Six years later, the same studies were performed, which showed that fasted GLP1 and GIP levels were both significantly lower at the follow-up studies (Pham et al., 2019). However, multiple other studies have shown increased incretin levels with age. One found significantly increased fasted GLP1 and CCK plasma levels in older adults, with total ghrelin and GIP levels unchanged compared to younger adults. In the same study, CCK, GIP, and GLP1 levels were all increased postprandially with age (Giezenaar et al., 2017). However, a follow-up to this study found fasting GLP1 decreased in older compared to young adults, but postprandial GLP1 increased in older adults (Giezenaar et al., 2020). Other studies found that postprandial GLP1 levels were increased in older adults; one study also showed postprandial PYY levels were increased while the other showed that postprandial GIP levels did not change with age (Crabtree et al., 2020; de Jesus Garduno-Garcia et al., 2018). A different study found increased GLP1 levels, lower acylated/deacylated ghrelin ratio, and decreased hunger in older adults after a high fat meal (Di Francesco et al., 2010). In a study looking at both humans and rats, older adults (greater than 65 years) had increased levels of circulating GLP1 when compared to controls (less than 65 years), while 20-month-old rats had higher levels of plasma GLP1 and GIP when compared to two-month-old rats (Wang et al., 2023). Notably, multiple other studies found no change in either fasted or postprandial GLP1 levels in older adults (Malafarina et al., 2013; Moss et al., 2012; Wysokinski et al., 2015).

Overall, the studies reviewed point to an increase in specific satiety hormones (CCK, PYY, and possible GLP1 and GIP) and a decrease in hunger hormones (GHRL) produced by EE cells with age, which may, in part, help explain the anorexia of aging. The altered production of these EE hormones can possibly be explained by changes in the number of EE cells, and specific EE cell types, with age. Similar to the studies examining hormone levels, many have sought to quantify changes in the number of EE cells with age and have produced varying results.

A murine study showed a reduction in ISCs and secretory progenitor cells in aged mice (17–24 months) compared to young mice (3–4 months), but the number of EE cells within intestinal crypts were similar (Mihaylova et al., 2018). Another mouse study found the opposite, showing older mice (18–24 months) had significantly more ISCs and EE progenitor cells, but no difference in CHGA expression in older versus younger (2–4 months) mice (Moorefield et al., 2017). However, two other mouse studies have found aging to increase the number of differentiated cells of the gut, finding more enterocytes, goblet cells, tuft cells, and EE cells within the villi of older mice (18 months and greater than 24 months) compared to young mice (2 months and 3–5 months) (Igarashi et al., 2019; Omrani et al., 2023). A human study looked at EE cell number in multiple age ranges and noted an increase in overall EE cell number in 20–29 year old adults compared to 1–2 year old children, although older patients in the study, up to 69 years of age, did not show any significant increase in EE cell number after 29 years (Sandstrom and El-Salhy, 1999a). This study also looked at numbers of specific EE cell types and found an increase in G and I cells in 60–69 year old adults compared to 20–29 year old adults, an increase in D cells in 40–49 year old compared to 20–29 year old adults, and an increase in EC cells in 20–29 year old adults compared to 1–2 year old children (Sandstrom and El-Salhy, 1999a). Interestingly, a recent paper compared jejunal organoids grown on monolayers from 10 to 22-week-old infants (n = 3) and those from 46 to 52 year old adults (n = 3) and found that the infant samples had an increased number of EE cells and increased expression of the EE cell markers *CHGA*, *CHGB*, and *SST* (Adeniyi-Ipadeola et al., 2024).

Looking at specific EE cell types, one mouse study reported a greater number of colonic GLP1-, PYY-, and 5-HT-producing cells in 12 and 24 month old mice compared to three month old mice (Sandstrom et al., 1998). In contrast, one human study of rectal biopsies from 20 subjects below 55 years of age and 20 above 55 years of age suggested that 5-HT- and PYY-expressing cells did not decline with age (Dunlop et al., 2004). Another study examining rectal biopsies found no differences between age groups and the number of GLP1-, PYY-, SST-, and 5-HT-positive cells (Sandstrom and el-Salhy, 1999b). In a study describe above, older adults (greater than 65 years old) were found to have increased numbers of total EE cells and GLP1-positive cells per crypt of the sigmoid colon and rectum (but not other parts of the colon) when compared to younger adults (Wang et al., 2023). This suggests that the age-related changes in EE cell composition may occur in some, but not all parts of the GI tract. In support of this, another study in humans suggested that the number of ileal EC cells increases with age, from 50 to almost 90 years of age (Yu et al., 2016).

Several studies have begun to find epigenetic regulators, pathways, and transcription factors that can alter ISC function and EE cell differentiation with age. One study using Drosophila at ages 5–8 days, 29–32 days, and 61–63 days found a progressive increase in the proportion of mature EE cells with age. Utilizing a multiomics approach, this phenotype was linked to altered Polycomb histone methylation activity, as inhibition of the Polycomb complex prevented the age-related increase in EE cell number (Tauc et al., 2021). This suggests an age-related decline in chromatin regulation in ISCs, which may be responsible for driving an increase in EE cells with age.

One important pathway that has been implicated in this age-related phenotype is Wnt signaling, which has been studied in multiple papers with different conclusions. One mouse study found that small intestine organoids from older mice (24 months old) showed significantly reduced *Chga* expression than organoids from young mice (2 months old), with higher expression of Wnt target genes (Cui et al., 2019). This suggests that Wnt signaling in ISCs increases with age, driving decreased differentiation in older mice. In contrast, another study suggested that Wnt signaling is reduced in older mouse ISCs and noted an increase in the number of Paneth and goblet cells in the small intestine from older mice, suggesting more secretory lineage differentiation (EE cells were not checked) (Nalapareddy et al., 2017). Another paper also noted a reduction in Wnt signaling in older human and mouse intestines, suggesting this was due to increased Paneth cell production of Notum (a Wnt inhibitor). This study found no difference in the number of crypt EE cells between young (3–9 months) and older (greater than 24 months) mice (Pentinmikko et al., 2019).

Another pathway that has been implicated is IFNγ signaling. One transcriptomic study found older mouse (18 months) ISCs have increased expression of secretory lineage markers compared to younger mouse (2 months) ISCs. Further, crypts from older mice had a greater proportion of EE cells, goblet cells, tuft cells, and enterocytes compared to younger mice (Omrani et al., 2023). This altered ISC differentiation was noted to be cell non-autonomous since intestinal organoids derived from older mice expressed less differentiated cell markers, including EE cell markers, compared to isolated crypts from older mice. The source of this differentiation phenotype was identified as increased IFNγ production from intestinal immune cells in older mice. To verify this, older adult organoids were treated with IFNγ, which increased differentiation of tuft and goblet cell markers (EE cell markers were not checked) (Omrani et al., 2023). Further, inhibition of IFNγ signaling using an anti-IFNγ antibody reversed this aging phenotype and brought the number of EE cells in older samples down to the number found in young samples (Omrani et al., 2023).

Another study implicated the transcription factor PDX1 as an important player in age-related EE cell changes. Older mice (12 months) were found to have increased number and activity of K cells when compared to younger mice (3–4 months). Interestingly, isolated K cells were noted to have increased *Pdx1* expression, with inhibition of this transcription factor in older mice reducing the number of K cells, *Gip* expression, and GIP secretion (Ikeguchi et al., 2018). This suggests that PDX1 plays a role in increased EE cell number and hormone production with age. Lastly, although Notch signaling plays a critical role in secretory lineage differentiation, the data regarding its role in EE cell differentiation with age is contested. A mouse study showed that older mouse ISCs have reduced *Notch1* expression; with this, there was an increase in *Atoh1* expression, suggesting increased secretory cell differentiation, but EE cell differentiation was not specifically evaluated (Nalapareddy et al., 2017). However, a study in Drosophila showed increased Notch signaling in 40-day-old ISCs compared to three-day-old ISCs with no change in the percentage of EE cells, although the total number of EE cells was increased in older intestines (Biteau et al., 2008).

It is clear the anorexia of aging is caused, at least partially, by an increase in satiety hormones and decrease in hunger hormones with age. Although it appears that these changes coincide with altered EE cell number, many of the reviewed studies have conflicting results. This may be secondary to variability in culturing techniques/analysis, area of the GI tract studied, differences between mouse and human GI tracts, and other long term health issues that can occur with age. Many of these variables are difficult to control for, especially in human studies, but for future studies, increasing the study size, analyzing multiple areas of the GI tract, and performing a thorough medical history will help better define the changes within the EE cell lineage with age.

## 6. Enteroendocrine cells and inflammatory bowel disease

Inflammatory bowel disease, e.g., Crohn’s Disease (CD) and Ulcerative Colitis (UC), are chronic and remitting disorders responsible for causing inflammation of the GI tract. IBD results from the interplay of genetic susceptibility and environmental exposure, which through a weakened epithelial barrier can elicit overt immune responses (Ramos and Papadakis, 2019). Although EE cells communicate directly with immune cells and neurons in health, their role in human IBD is not well understood (Bogunovic et al., 2007; El-Salhy et al., 2017b; Nwako and McCauley, 2024; Worthington et al., 2018; Yu et al., 2020). As chronic inflammation can lead to changes in the composition of the intestinal epithelium, including EE cell number (Fig. 5), a deeper understanding of EE cell differentiation and function in the context of intestinal inflammation may lead to a mechanistic understanding of the non-inflammatory-based symptoms often observed in patients with IBD, including changes in motility, anorexia, and malabsorption.

While the role of EE cells in IBD pathogenesis is not fully understood, the impact of inflammation in disrupting the GI epithelium during IBD pathogenesis has been broadly demonstrated (Ramos and Papadakis, 2019). Interestingly, phenotypic changes seen due to intestinal inflammation may reflect how the intestinal epithelial barrier can adapt from previous exposure to specific immune events (Ordovas-Montanes et al., 2020). Alterations in EE cell numbers and hormonal secretion have been reported in both patients with IBD and in animal models of colitis (El-Salhy et al., 1997, 2017a; El-Salhy and Hatlebakk, 2016). Immunohistochemical analysis of IBD biopsies has shown an increase in colonic CHGA and EC cells in both UC and CD patients (El-Salhy et al., 1997). These findings were replicated in the model of dextran sodium sulfate (DSS)-induced colitis, with an increase in colonic CHGA-, PYY-, and 5-HT-positive cells, which correlated with an increase in specific immune cell types, including T lymphocytes and macrophages, when compared to control animals. Interestingly, an inverse correlation of EE cell markers and inflammation was seen in a different model, trinitrobenzene sulfonic acid (TNBS)-induced colitis, which demonstrated lower numbers of colonic CHGA- and 5-HT-positive cells, correlating with an increase in immune cell populations compared to controls (El-Salhy and Hatlebakk, 2016). As DSS and TNBS utilize different mechanisms to induce colitis, these findings suggest an important role of inflammation in mediating the epithelial composition in mice and patients with colitis. A recent study examining murine EE cells in the setting of DSS-induced colitis found a significant reduction in EE cell density within the small intestine, as revealed by immunohistochemical and gene expression analysis for CHGA, and a significant reduction in key transcription factors involved in EE cell differentiation such as *Atoh1*, *Neurog3*, and *Neurod1* (Raouf et al., 2024). This study suggests that location within the GI tract heavily influences how EE cell differentiation changes in response to DSS exposure.

It is known that EE cell markers, such as paired-like homeobox 2b (PHOX2B) and ubiquitination factor E4A (UBE4A), have been identified by genome-wide association studies in patients with IBD, suggesting an important role for EE cells in the pathogenesis of IBD (Rioux et al., 2007). Moreover, EE cells express toll-like receptors and can respond to luminal changes from microbial metabolites with hormone secretion (Bogunovic et al., 2007). EE cells have been shown to indirectly, through hormone and even cytokine secretion, interact with innate and adaptive immune cells, *in vitro* (Worthington et al., 2018; Yu et al., 2020). Specifically, reports show that the EE cell hormones CCK, GIP, and 5-HT have direct effects on T cell activation and differentiation (Efimova et al., 2021; Leon-Ponte et al., 2007; Zhang et al., 2014). For instance, CCK can indirectly promote the T-helper (Th) 2 and regulatory T cell (Treg) phenotype, *in vitro*, via activation of MAPK signaling (Zhang et al., 2014). Furthermore, 5-HT has previously been shown to activate T cell responses *in* vitro (Leon-Ponte et al., 2007). Accordingly, studies have shown that treatment of patients with neuroendocrine tumors with the SST-analog octreotide results in a decrease in the number of circulating T regs (von Arx et al., 2020). Importantly, octreotide is often used for the management of diarrhea in patients with IBD and short bowel syndrome and GIP analogs are increasingly being used in the management of obesity, which can also affect patients with IBD. Whether these medications can affect inflammation in patients with IBD remains unclear. Further understanding of how EE cells and their hormones modulate immune responses may help explain potential side effects seen with these medications in patients with IBD, in addition to unraveling novel therapeutic approaches for intestinal inflammation.

It is known that intestinal inflammation alone does not explain the entirety of IBD-related symptoms, as over one-third of patients still experience symptoms even in the absence of inflammation (Halpin and Ford, 2012). Thus, it is possible that dysregulation of EE cell differentiation and function in patients with IBD could underlie symptomatology via non-inflammatory pathways. Indeed, levels of different EE cell hormones were found to be distinct between patients with IBD and healthy individuals. For instance, higher GLP1 secretion has been reported in patients with ileal CD compared to controls, resulting in an overall 52 % increase in insulin secretion in this patient population. Interestingly, these results were independent of disease activity or prior history of ileal resection (Bendet et al., 2004). Elevation in GLP1 levels have also been described in patients with UC, in a study comparing different EE cell hormone levels and gastric emptying in patients with IBD compared to healthy controls. In this same study, CD patients also demonstrated a three-fold increase in postprandial CCK levels compared to healthy controls, which correlated with delayed gastric emptying and is believed to be an inflammation-independent mechanism that might explain persistent upper GI symptoms in this population (Keller et al., 2009). Finally, significant changes in circulating levels of CHGA, GHRL, GIP, MLN, PYY, and SST have been reported in patients with IBD and have been proposed to modulate IBD symptoms such as decreased appetite, fatigue, and pain (Binimelis et al., 1987; Karmiris et al., 2006; Moran et al., 2013; Strid et al., 2013; Ter Beek et al., 2008). These findings highlight the important role EE cells may play in both inflammation- and non-inflammation-based symptoms in IBD. Further understanding of EE cell differentiation (including alterations of important transcription factors mentioned above) and function may open novel therapeutic approaches to improve the care of patients with IBD.

## 7. Enteroendocrine cells in obesity and diabetes mellitus

Due to the role of EE cell hormones in regulating appetite and insulin production, EE cell function is intertwined with the pathophysiology of both obesity and T2DM. This is highlighted by the loss of the incretin effect in patient with T2DM (Nauck and Meier, 2016); however, the changes in production and response to EE cell hormones in obesity and T2DM expand well beyond GIP and GLP1. Thus, it is no surprise that there have been many studies and published reviews that examine EE cell hormones in obesity and T2DM, with the consensus that the levels of the majority of anorexigenic and incretin hormones are decreased (in both the fasted and postprandial state). However, there are some rare differences in EE cell hormone secretion between obesity and T2DM that are reviewed elsewhere, as well as some conflicting data (Atanga et al., 2023; Farhadipour and Depoortere, 2021; Gribble and Reimann, 2019; Koliaki et al., 2020; Nauck and Meier, 2016; Ricardo-Silgado et al., 2021). Below we will review some of the more comprehensive and recent studies looking at human EE cell hormone levels and cell numbers in obesity and T2DM (Fig. 5).

Examining obesity first, a study comparing 20 individuals with obesity to 19 healthy-weight controls showed decreased secretion of GLP1 20 min after a liquid meal without a significant decrease in GHRL at 1 h after the same meal (Carroll et al., 2007). Another study evaluated fasted and postprandial levels of CCK, GAST, GHRL, and PYY in women with moderate (BMI>35kg/m2, n = 12) and morbid obesity (BMI>40mg/m2, n = 17) compared to eight healthy-weight controls. In the fasted state, total and acylated GHRL were significantly decreased in the moderate obesity group compared to controls while PYY and CCK were significantly decreased in the morbidly obese group compared to controls. Postprandially, individuals with moderate obesity had an appropriate decrease in serum total GHRL levels, which was not seen in the morbidly obese group. Interestingly, neither of the groups with obesity had a decrease in their acylated GHRL levels, which would suggest continued hunger after eating. This is supported by the post-prandial PYY data, which showed decreased production in both groups. All groups showed an increase in CCK serum levels 30 min after eating, but levels in the group with morbid obesity were lower than in both the group with moderate obesity and the healthy-weight control. Finally, postprandial GAST levels were significantly decreased in both those with moderate and morbid obesity (Zwirska-Korczala et al., 2007).

A more recent study looked at fasting GIP, GLP1, and PYY levels in 29 individuals with overweight (one with T2DM), 161 individuals with obesity (five with T2DM), and 15 healthy-weight controls, and showed decreased levels of serum PYY in the groups with overweight and obesity, while the group with overweight showed an increased level of fasting GIP, and the group with obesity demonstrated a significantly higher level of GLP1. Interestingly, if run on a different assay, fasted GLP1 levels showed no change between the three groups (Bannon et al., 2024). It is worth mentioning that GIP secretion in obesity and T2DM does not act similarly to its incretin partner GLP1; GIP secretion has been found to be increased in the setting of high-fat diet induced-obesity (Holst and Rosenkilde, 2020). Additionally, a meta-analysis of 23 studies showed that postprandial GIP secretion was unchanged in patients with T2DM when compared to healthy-weight controls, but that increased BMI was associated with increased GIP secretion and that worsening diabetes control (higher hemoglobin A1c) and age were associated with decreased GIP secretion (Calanna et al., 2013). Oxyntomodulin and glicentin levels are also altered in the setting of obesity and T2DM. In a small study, patients with T2DM and obesity, but not those with only obesity, produced less oxyntomodulin and glicentin during an oral glucose tolerance test when compared to healthy controls (Wewer Albrechtsen et al., 2016).

Moreover, another study evaluated fasted EE cell hormone levels in a pediatric population (Giannini et al., 2022). Comparing healthy-weight controls (n = 30) to children with obesity with varying degrees of metabolic syndrome (n = 30 for each group) showed that fasted levels of GHRL and GLP1 were unchanged in children with obesity without metabolic syndrome, while obestatin, an anorexigenic hormone, was significantly elevated. Having obesity with metabolic syndrome led to decreased plasma levels of fasted GHRL and GLP1 and increased levels of obestatin when compared to healthy-weight controls. Overall, these studies, and those reviewed previously, suggest that the CCK, GLP1, and PYY levels in those with obesity and/or T2DM are typically decreased in the fasted and postprandial state, while GHRL does not decrease in the postprandial state. In addition, GIP levels can be unchanged, increased, or decreased depending on the metabolic state of the individual. Data also suggest that oxyntomodulin and glicentin are negatively regulated in T2DM while obestatin appears to have increased expression in the setting of obesity. This suggests that the ability of obestatin to induce appetite suppression is not as substantial as other anorexigenic hormones or that there is obestatin resistance in the face of obesity.

This altered EE hormone secretion in patients with obesity/T2DM has multiple possible causes, including altered nutritional stimuli, altered downstream target response, and altered ISC differentiation. In fact, multiple studies have found altered numbers of some EE cells in patients with obesity and T2DM, which suggests changes with EE cell differentiation. In a study comparing patients with new-onset T2DM to healthy controls, it was noted that new-onset T2DM was associated with increased postprandial secretion of GLP1, with no change in GIP secretion. With this, it was noted that patients with new-onset T2DM had significantly more L cells in the duodenum compared to controls (Theodorakis et al., 2006). In a more in-depth study, biopsies were taken from 16 different portions of the intestine (duodenum to rectum) in BMI-matched, non-obese patients with T2DM (average of five years duration) and healthy controls. This study noted an overall decrease in CHGA-positive EE cells in the small intestine of patients with T2DM, with no significant change to the number of K and L cells throughout the GI tract, although mRNA expression of *GIP* was increased in both the large and small intestines of the T2DM group (Jorsal et al., 2018). In follow-ups to this study, there was an increased number of colonic CCK-positive cells (no change in the small intestine) in patients with T2DM, although there were less I cells in the colon compared to the small intestine, and no difference between SCT-positive cells between healthy controls and patients with T2DM (Gilliam-Vigh et al., 2021, 2023). Along with this, another study found no difference in ileal GLP1-positive cell density when comparing patients with and without T2DM (Kampmann et al., 2016); however, a different study showed that in older individuals, having T2DM leads to decreased GLP1 secretion and fewer colonic GLP1 positive cells (Wang et al., 2023).

Interestingly, jejunal L cell number was significantly decreased in patients with obesity and T2DM when compared to patients with obesity without T2DM (Osinski et al., 2021), while another group, also comparing jejunal samples of patients with obesity with and without T2DM, noted no differences between K and L cells but found an increased number of N cells in patients with T2DM (Ferreira et al., 2023). Another study found no difference in the number of colonic I, EC and L cells (based on PYY expression) in a mixed group of patients with overweight and obesity (BMI greater than 25 kg/m^2^) compared to heathy-weight individuals (Baumard et al., 2021). Murine studies have also been used to evaluate EE cells during obesity and T2DM. One study of obesity (12 weeks of a high-fat, high-sugar diet) showed fewer total EE cells in the duodenal villi, decreased number of EC cells in duodenal crypts, increased number of X/A cells in duodenal villi, and increased L cells in ileal villi. Suggesting a possible differentiation phenotype, increased numbers of goblet cells and enterocytes were also seen. Using single cell RNA-sequencing, it was also noted that the high-fat, high-sugar diet reduced the number of *Sox4*-positive progenitor cells but increased the number of *Ngn3*-positive EE progenitor cells (Aliluev et al., 2021). Another mouse model noted increased numbers of both gastric GHRL- and GAST-positive cells after both three weeks and six months of high fat diet exposure (Widmayer et al., 2015), while eight weeks of high fat diet increased the number of colonic, but not duodenal, EC cells, with an increase in circulating 5-HT levels (Martin et al., 2020). Further, in patients with obesity, high fat diet led to an increased number of jejunal GLP1-positive cells; this was also seen in mouse jejunum and colon, with no change in the number of PYY-positive cells. It was also noticed that after eight weeks of high fat diet, expression of *Foxa1*, *Foxa2*, *Isl1*, and *Pax6* were mildly increased in the jejunum and colon compared to control mice (Aranias et al., 2015).

A group of studies utilized bariatric surgery, which is well known to alter the production of multiple EE cell hormones (Moffett et al., 2021; Nielsen et al., 2020), to analyze the number of EE cells both before and after treatment. A large study, including 24 healthy-weight controls, 33 patients with obesity before sleeve gastrectomy, and 27 patients with obesity three months post-operative (no patients had T2DM), evaluated CHGA, CCK, GHRL, GLP1, GLP2, and PYY expression within the duodenum and stomach (Wolnerhanssen et al., 2017). Prior to surgery, patients with obesity had significantly fewer CHGA-, CCK- GHRL-, GLP1-, GLP2-, and PYY-positive cells. In all EE cell types analyzed, sleeve gastrectomy significantly restored all cell numbers close to the healthy-weight control baseline (Wolnerhanssen et al., 2017). Using Western blot analysis on intestinal epithelial tissue, they also found that HES1 levels were increased in patients with obesity and were reduced after bariatric surgery while levels of ATOH1, NEUROD1, and NEUROG3 were all reduced in patients with obesity and increased to healthy-weight control levels after bariatric surgery. They also noted other mature intestinal cell numbers were altered in patients prior to sleeve gastrectomy, including Paneth cells, which were significantly decreased compared to healthy-weight controls, and goblet cells, which were significantly increased compared to healthy-weight controls. Interestingly, sleeve gastrectomy reduced the number of goblet cells significantly but was unable to increase the number of Paneth cells back to control levels (Wolnerhanssen et al., 2017). In a mouse study using vertical sleeve gastrectomy, postprandial plasma levels of GLP1 and PYY were increased four weeks post-operatively, with a corresponding increase in jejunal GLP1-positive cells and duodenal and ileal PYY-positive cells (Kim et al., 2022).

An additional study compared different EE cells in patients with obesity before Roux-en-Y gastric bypass (RYGB, jejunal tissue) and four months after RYGB from different parts of the intestine, including the Roux (alimentary) limb, biliopancreatic (BP) limb, and the common limb (Rhee et al., 2015). Splitting the patients into two groups based on T2DM, this study found increased CCK-, GIP-, and GLP1-expressing cells in all three limbs after RYGB surgery in both groups. Only patients without T2DM showed a post-operative increase in PYY-positive cells. Interestingly, gene expression was also evaluated in this study and was not entirely consistent with protein expression; although *GCG* expression was increased after RYGB, there was no change in *PYY* or *CCK* expression and *GIP* expression was actually decreased post-operatively (Rhee et al., 2015). A more recent study examined the number of EE cells before and after RYGB in rats fed either normal chow or a high-fat diet. With the caveat that there were only two rodents per group, this study showed no significant differences in GIP- and GLP1-positive cells after RYGB in either diet group. Post-operatively, GLP2-positive cells were found to be significantly increased, while, interestingly, PYY-positive cells were decreased, but only in the Roux limb of normal chow fed rats (Sridhar et al., 2023).

Cystic fibrosis-related diabetes (CFRD) is another entity that shows altered EE cell hormone production. Due to rapid improvements in treatment, patients with CF are now living well into adulthood, leading to an increased prevalence of extra-pulmonary manifestations of CF, including CFRD (Ticona et al., 2023). CFRD is believed to have multiple possible causes, including endocrine and exocrine pancreatic dysfunction, genetic predisposition, medical treatments, and dysregulated EE cell hormone production (Granados et al., 2019; Moheet and Moran, 2022). In fact, similar to T2DM, there is a loss of the incretin effect in patients with CF and CFRD, and multiple studies have shown that levels of serum GIP are decreased in patients with CF and CFRD when compared to healthy controls (Adrian et al., 1980; Frost et al., 2019; Hillman et al., 2012; Knop et al., 2007; Perano et al., 2014; Sheikh et al., 2017). Different from T2DM, the majority of studies suggest that GLP1 levels are unchanged in patients with CF and CFRD, with only two studies showing a decrease in GLP1 secretion (Hillman et al., 2012; Kuo et al., 2011). Even though CFTR is expressed in ISCs (Strong et al., 1994; Strubberg et al., 2018), only one study has examined EE cell number in CF (Kelly et al., 2022). CFTR knockout mice were found to have decreased numbers of ileal CHGA-, GLP1-, and GLP2-positive EE cells. Further, the number of ileal ISCs and goblet cells was increased while the number of Paneth cells was decreased. Analysis of just one patient with CF, compared to seven healthy controls, showed a similar pattern of decreased ileal EE and Paneth cells, and increased ileal ISCs and goblet cells (Kelly et al., 2022).

Lastly, a significant difference in EE cell number has been noted in the setting of T1DM (Bharadiya et al., 2024). Differentiation of duodenal organoids derived from two patients with T1DM and three healthy controls showed an increase in the number of CHGA-positive cells per organoid, as well as increased expression of *GHRL* and *LMX1A* (as a marker of EC cells) in both differentiated and undifferentiated states. Interestingly, *PYY* levels were decreased in the undifferentiated state and not significantly different from controls upon differentiation (Bharadiya et al., 2024). These findings contrast with those seen in T2DM, which could be secondary to the different causes of these diseases, as well as the small sample size.

Taken together, these studies suggest that the observed changes in the number of EE cells mirror the changes that are seen in hormone production in patients with obesity and T2DM. It is unclear what causes this change in EE cell number, but it is possible that ISC differentiation is altered, especially since some studies have shown changes in other intestinal lineages, along with decreased expression of key secretory and EE cell transcription factors that improve following bariatric surgery. There is also some conflicting data among the reviewed studies in terms of hormone production and EE cell number, which could stem from location of GI tract analyzed, species differences, duration of DM, glycemic control, and weight status (overweight, obese, morbidly obese). Future studies should ensure that these variables are highlighted so to not confound any results.

## 8. Enteroendocrine cells as a source of insulin production

Although they are not FDA-approved treatments like GLP1/GIP receptor agonists, cell-based therapies to treat, and possibly cure, diabetes mellitus (including both T1DM and T2DM), have seen incredible progress over the last decade. The use of induced pluripotent stem cells (iPSCs) to derive insulin-producing beta-like cells has been refined by multiple labs and has been shown to reduce, and sometimes remove, the need for insulin in mice, and in a small number of humans with DM (Ghoneim et al., 2024; Maxwell and Millman, 2021; Wang et al., 2022). An alternative strategy pursued by investigators has been to directly target EE cells to produce endogenous insulin (Fig. 5). The rationale for this approach is based on the facts that EE cells and pancreatic beta cells (1) share a common developmental origin (McGrath and Wells, 2015), (2) express multiple common transcriptional regulators critical for insulin production, including PDX1, NEUROD1, and NEUROG3 (Balakrishnan et al., 2020), and (3) utilize common mechanisms needed to process propeptides (e.g., prohormone convertases), sense glucose (e. g., GLUT2), and store and secrete peptide hormones (Encina et al., 2011). These similarities are underscored by a recent study using human fetal intestine that showed a small minority of K and L cells that express and produce insulin; however, the insulin-producing ability from these cells was lost postnatally (Egozi et al., 2021).

To establish that EE cells can produce and secrete insulin in response to glucose, studies have focused on driving insulin expression in K and L cells. For example, expression of insulin under the control of the GIP and GLP1 promoters in the STC1 EE cell line led to secretion of functional insulin in response to glucose exposure (Encina et al., 2011). In murine studies, these transgenic cells were able to regulate blood glucose levels in streptozotocin (STZ)-treated mice, maintaining euglycemia after native beta cell destruction. Furthermore, these mice did not experience persistent hypoglycemia, suggesting that these transgenic K cells were secreting insulin appropriately (Cheung et al., 2000; Han et al., 2007; Mojibian et al., 2016). Interestingly, the expression of insulin from K cells in a model of autoimmune DM (using NOD mice) was found to be protective against developing DM, with less than 20 % of transgenic mice developing DM after 32 weeks of age (compared to over 80 % developing DM in the non-transgenic group), irrespective of pancreatic insulin production. Notably, these cells also appeared to avoid the immune response, with no obvious intestinal infiltration of immune cells and decreased production of the inflammatory cytokine INFγ (Mojibian et al., 2014).

Moving upstream, multiple studies have looked at why K and L cells do not express insulin postnatally, especially as they express most of the machinery necessary to produce and secrete insulin. One study comparing mature EE cells from the STC1 cell line and insulin-producing cells from the beta cell line MIN6 showed that EE cells had comparable expression levels of genes required for insulin secretion. However, gene expression of the transcription factors *Mafa*, *Nkx6-1*, and *Pdx1* were all expressed at much higher levels in MIN6 cells. Multiple mature EE cell types also showed increased methylation at the *Ins2* promoter, which would suppress insulin gene expression (Ryu et al., 2018). Interestingly, using STC1-derived K cells, insulin production could be induced by over-expression of *Neurog3* and *Nkx6-1*; however, these cells had reduced insulin secretion in response to glucose compared to a beta cell line and were unable to rescue an STZ model of DM (Lee et al., 2014). Another set of studies found that overexpression of *Pdx1*, *Mafa*, and *Neurog3* (PMN) in EE cells from stomach, duodenum, and jejunum could induce insulin production (mouse and human) both *in vitro* and *in vivo* (Ariyachet et al., 2016; Chen et al., 2014; Huang et al., 2023). One study showed that persistent activation of whole body PMN induced symptomatic hypoglycemia *in vivo* secondary to insulin production in the intestine; once PMN overexpression was stopped, the remaining insulin-producing cells were able to regulate blood glucose levels without hypoglycemia (Chen et al., 2014). A second study, however, suggested that persistent activation of EE cell-specific PMN in the setting of STZ-induced DM could maintain euglycemia over 24 weeks, suggesting appropriately regulated insulin production and secretion (Ariyachet et al., 2016). One study identified the EE cells from antral stomach as being the most transcriptionally similar to pancreatic beta cells when compared to duodenal and colonic EE cells, leading to the utilization of adult human gastric ISCs to derive insulin-producing EE cells (Huang et al., 2023). Using sequential expression of PMN (NEUROG3 followed by both PDX1 and MAFA), human gastric organoids were derived that could produce insulin in response to glucose and GLP1 receptor agonists, *in vitro*. When these organoids were implanted under the kidney capsule of immunodeficient NSG mice treated with STZ, they induced, and maintained, an almost complete resolution of hyperglycemia as evidenced by normalization of fasting blood glucose levels and glucose tolerance tests (Huang et al., 2023).

In parallel, experiments have been published demonstrating that deletion of *FOXO1* can serve as an additional mechanism to induce insulin production from EE cells. *Foxo1* deletion in NEUROG3-positive cells led to the production of insulin-producing cells in the small and large intestine, which was also seen in adult mice when using an inducible Neurog3-Cre system. In the setting of STZ, *Foxo1* deletion induced insulin production from EE cells that was able to improve, but not completely correct, fasting blood glucose levels and glucose tolerance tests, without inducing hypoglycemia (Talchai et al., 2012). A subsequent study showed that *FOXO1* deletion in human intestinal iPSC-derived organoids could also induce insulin production in response to high glucose, arginine, and potassium chloride, *in vitro* (Bouchi et al., 2014). Taking a drug-treatment approach, three recent studies examined oral agents aimed at inhibiting FOXO1 and other targets to induce EE cell insulin production *in vivo* (Du et al., 2022; Kitamoto et al., 2022; Lee et al., 2022). Using the STZ model, mice with diabetes were given two different FOXO1 inhibitors, FBT374 and FBT432. FBT374 was able to improve fasting blood glucose levels after only two days of treatment. After six days of treatment, both fasting blood glucose levels and glucose tolerance tests improved to levels that were not significantly different from wild type mice. In comparison, although 12 days of treatment with the second small molecule FBT432 improved fasting blood glucose levels and glucose tolerance tests in STZ-treated mice, the results were not as robust as with FBT374 or the previously described *Foxo1* knockout mice (Lee et al., 2022). To improve upon this, an oral Notch inhibitor targeting gamma secretase was added in combination with FBT432, which again induced insulin-producing EE cells that appeared to reverse the diabetes phenotype in STZ-treated mice (Lee et al., 2022). Similar findings were noted in an additional study looking at the FOXO1 inhibitor FBT10 and a gamma secretase inhibitor given to a genetic mouse model of T1DM (Kitamoto et al., 2022). Finally, triple therapy using the FOXO1 inhibitor FBT10, a gamma secretase inhibitor, and a TGFβ inhibitor for five days improved glucose tolerance tests in NOD mice and induced insulin production and secretion in human primary duodenal organoids as part of a larger differentiation protocol (Du et al., 2022).

Together, these studies represent more than a decade of impressive advancements aimed at utilizing EE cells to treat DM. Insulin production from EE cells has numerous advantages, including ease of access to cells of interest (either through endoscopy or via orally administered medications) and possibly immune evasion, which would be critical for patients with autoimmune-induced T1DM. Much work still needs to be done, however, including understanding the immunoreactivity of implanted PMN cells and if it would be possible to place them near the intestinal epithelium to possibly avoid immune detection. Further, although orally administered medications would be the most appealing method to induce EE cells to produce insulin, it is unclear how inhibition of FOXO1, Notch signaling, and TGFβ would alter whole body organ function in the long-term. Regardless of these caveats, these two areas of research (PMN cells and FOXO1 inhibition) are set to propel EE cells further into the forefront of DM treatment, highlighting the importance of these cells in glucose homeostasis.

## 9. Conclusion

EE cell differentiation is a complex process that is driven by transcription factors, signaling pathways, normal human physiology, and disease states. As our culturing and multiomics tools become more advanced, our understanding of EE cell differentiation grows, but there continues to be much that is unknown. The number of critical transcription factors that regulate EE cell differentiation continues to increase, and many of the known factors can function upstream in ways not always appreciated. Using techniques focusing on chromatin accessibility and RNA expression, the field can finally map how different transcription factors interact with each other within the EE cell lineage to guide maturation to a specific hormone producing cell.

Further, aging and multiple disease states can regulate EE cells as well, but it continues to be unclear if this is specifically at the hormonal level or if these states alter ISCs. With our improvements in organoid culturing techniques and ability to assay individual cells at the RNA, chromatin, and protein levels, we are at the cusp of truly understanding the effects health and disease have on the human EE cell, and how the EE cell can alter health and disease.

Understanding EE cell differentiation and function is critical for using them as medical treatment. We discussed the use of EE cells for the possible treatment of T1DM, but the potential therapeutic benefits extend well beyond just T1DM. Designing medications that can alter either EE cell differentiation or EE cell hormone production will have large implications for obesity, T2DM, malnutrition/frailty, IBD, and cystic fibrosis. As our knowledge pertaining to EE cell differentiation grows, so does the potential for helping people with illnesses that can affect all stages of life.

## Figures and Tables

**Table 1:** Signaling Pathways that Regulate Enteroendocrine Differentiation

Inhibits EE Differentiation	Promotes EE Differentiation
Canonical WNT	BMP
EGF	EREG
Endocannabinoid	Hippo?
Hedgehog?	Non-canonical Wnt/PCP
JNK/ERK	NRG1
mTORC1	P38 MAPK
Notch	

Signaling pathways involved in inhibiting (left) and promoting (right) enteroendocrine differentiation. The question marks denote pathways that have not been robustly studied within the enteroendocrine lineage.

## Data Availability

No data was used for the research described in the article.
